# Inner membrane fusion mediates spatial distribution of axonal mitochondria

**DOI:** 10.1038/srep18981

**Published:** 2016-01-08

**Authors:** Yiyi Yu, Hao-Chih Lee, Kuan-Chieh Chen, Joseph Suhan, Minhua Qiu, Qinle Ba, Ge Yang

**Affiliations:** 1 Department of Biomedical Engineering, Carnegie Mellon University, Pittsburgh, PA 15213; 2 Center for the Neural Basis of Cognition, Carnegie Mellon University, Pittsburgh, PA 15213; 3 Department of Biological Sciences, Carnegie Mellon University, Pittsburgh, PA 15213; 4 Department of Computational Biology, Carnegie Mellon University, Pittsburgh, PA 15213.

## Abstract

In eukaryotic cells, mitochondria form a dynamic interconnected network to respond to changing needs at different subcellular locations. A fundamental yet unanswered question regarding this network is whether, and if so how, local fusion and fission of individual mitochondria affect their global distribution. To address this question, we developed high-resolution computational image analysis techniques to examine the relations between mitochondrial fusion/fission and spatial distribution within the axon of Drosophila larval neurons. We found that stationary and moving mitochondria underwent fusion and fission regularly but followed different spatial distribution patterns and exhibited different morphology. Disruption of inner membrane fusion by knockdown of dOpa1, Drosophila Optic Atrophy 1, not only increased the spatial density of stationary and moving mitochondria but also changed their spatial distributions and morphology differentially. Knockdown of dOpa1 also impaired axonal transport of mitochondria. But the changed spatial distributions of mitochondria resulted primarily from disruption of inner membrane fusion because knockdown of Milton, a mitochondrial kinesin-1 adapter, caused similar transport velocity impairment but different spatial distributions. Together, our data reveals that stationary mitochondria within the axon interconnect with moving mitochondria through fusion and fission and that local inner membrane fusion between individual mitochondria mediates their global distribution.

Mitochondria are essential organelles of eukaryotic cells, serving a wide variety of important functions that include energy production, metabolic regulation, and stress response^1^,^2^. Through fusion and fission as well as transport and anchoring, individual mitochondria work together by forming a dynamic, interconnected, and spatially distributed network to respond to changing needs at different intracellular locations^3^. Significant advances have been made in identifying and characterizing the molecular machineries of mitochondrial fusion and fission^3^,^4^ as well as transport and anchoring^5^. However, our understanding of how the mitochondrial network operates spatially at the systems level remains limited. A fundamental yet unanswered question regarding the mitochondrial network is whether, and if so how, local fusion and fission of individual mitochondria affect their global distribution.

Neurons provide a powerful model system to answer this question because of their polarized structure and their critical dependence on the mitochondrial network for survival and function^6^,^7^,^8^,^9^,^10^,^11^. The long and thin axon, in particular, provides a simplified setting for high-resolution quantitative analysis of relations between local fusion/fission and global spatial distribution of the mitochondrial network. Several lines of data suggested connections between mitochondrial fusion/fission and spatial distribution in neurons. First, mitochondrial morphology and distribution in the axon changed simultaneously in response to excitatory and inhibitory stimuli^10^,^12^,^13^ and demyelination^14^,^15^. Second, mutations of mitochondrial outer membrane fusion protein Mfn2^16^ or inner membrane fusion protein OPA1^17^ changed both morphology and distribution of axonal mitochondria and caused neurodegeneration. Knockdown of OPA1 also changed morphology and distribution of dendritic mitochondria^18^. Third, recent studies started to reveal direct interactions between molecular machineries of mitochondrial fusion/fission and molecular machineries of mitochondrial transport^19^,^20^,^21^. Because spatial distribution of mitochondria is mediated directly by their transport and anchoring, mitochondrial fusion/fission and spatial distribution may be connected through interactions between their molecular machineries. Overall, these studies provided indirect evidence of connections between mitochondrial fusion/fission and spatial distribution. However, direct investigation of such connections remains lacking.

To directly and quantitatively analyze relations between mitochondrial fusion/fission and spatial distribution, we developed high-resolution computational image analysis techniques to track movement and morphological changes of individual mitochondria and to characterize their fusion/fission and spatial behavior. Performance of our computational image analysis technique for mitochondrial fusion/fission detection was validated independently by a photobleaching and fluorescence recovery assay. Using our computational image analysis techniques, we analyzed in high-resolution relations between fusion/fission and spatial distribution of mitochondria within the axon of motor neurons in Drosophila third instar larvae under normal conditions and knockdown of dOpa1, the Drosophila ortholog of human OPA1^22^. Knockdown of dOpa1 disrupted inner membrane fusion, allowing us to test specifically whether, and if so how, perturbation of the inner membrane fusion of mitochondria would affect their spatial distribution.

We first examined fusion/fission and spatial distribution of mitochondria within the axon of wild-type larval motor neurons. We found that stationary mitochondria underwent fusion and fission regularly with moving mitochondria. But the spatial distributions and morphology of these two groups of mitochondria were significantly different. Knockdown of dOpa1 caused a dramatic imbalance between fusion and fission, which resulted not only in an overall increase in spatial density of stationary and moving mitochondria but also in differential changes of their spatial distributions and morphology. Knockdown of dOpa1 also impaired transport of mitochondria. However, the changes to the spatial distributions of axonal mitochondria under dOpa1 knockdown were caused primarily by disruption of inner membrane fusion because knockdown of Milton, a mitochondrial adaptor for kinesin-1^23^,^24^, resulted in similar impairment of mitochondrial transport velocity but significantly different mitochondrial distribution patterns. Impairment of mitochondrial transport under dOpa1 knockdown played a secondary role in changing the spatial distribution of mitochondria. Together, our data provides novel insights into the relations between local fusion/fission and global spatial distribution of the mitochondrial network.

## Results

### Regular fusion and fission between stationary and moving mitochondria within the axon identified by computational image analysis

To investigate the relations between mitochondrial fusion/fission and spatial distribution, we first examined fusion and fission of axonal mitochondria within segmental nerves of Drosophila third instar larvae. We used a Gal4 strain SG26-1 to drive cell-specific expression of UAS-mito-GFP, which allowed us to image mitochondria in a single axon. Time-lapse movies were collected at 30 frames per minute for 40 minutes each. We identified fusion and fission in each time-lapse movie by tracking movement and morphological changes of individual mitochondria (supplementary Fig. S1) and checking their size changes using computational image analysis (see Materials and Methods). Potential fusion/fission events identified based on mitochondrial size changes were further visually verified to minimize false detection (see Materials and Methods).

**Figure 1:**
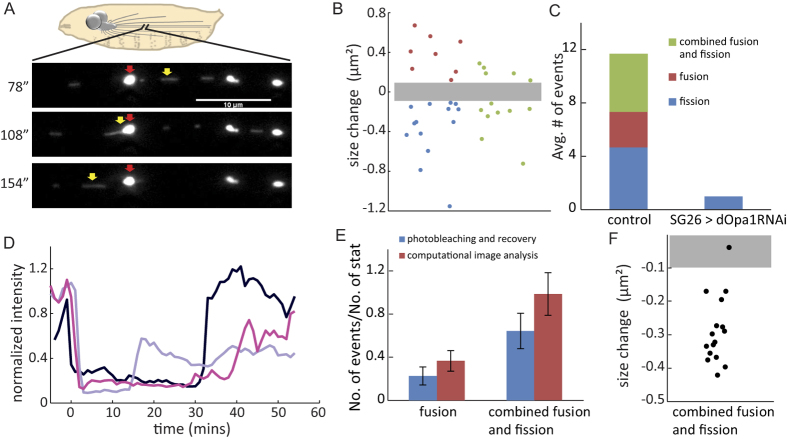
Identification of fusion and fission of axonal mitochondria by computational image analysis or photobleaching and fluorescence recovery. (**A**) A representative example of combined fusion and fission between a stationary mitochondrion (red arrow) and a moving mitochondrion (yellow arrow) within the axon of a Drosophila third instar larva. The size of the moving mitochondrion was increased by 0.25 μm^2^ after the combined fusion and fission. Scale bar: 10 μm. (**B**) Mitochondrial size changes in detected fusion (red), fission (blue), and combined fusion and fission (green) events in control larvae. The gray bar indicates the detection threshold (0.1 μm^2^). (**C**) Average number of fusion and fission events within 40 minutes of imaging in an ROI ~80 μm in length in axons of control and dOpa1 knockdown larvae. Control (n = 3): fusion = 2.7 ± 0.5, fission = 4.7 ± 2.5, and combined fusion and fission = 4.3 ± 1.7. SG26 > dOpa1RNAi (n = 4): fusion = 0, fission = 1 ± 0.7, and combined fusion and fission = 0. (**D**) Representative intensity time series of three mitochondria undergoing fluorescence recovery after photobleaching. Time 0 indicates the end of photobleaching. (**E**) Number of fusion and fission detected using computational imaging analysis (3 experiments) versus photobleaching and fluorescence recovery (5 experiments): The number of events was normalized by the number of stationary mitochondria in the ROI. (**F**) Size changes of moving mitochondria in combined fusion and fission events identified by intensity recovery. Out of 17 detected events, 16 (94.1%) exhibited significant size change.

We identified three types of events: fusion, fission, and combined fusion and fission (see Materials and Methods, Fig. 1A; supplementary Fig. S2-S4; supplementary Movies S1-S5). Mitochondrial size increases in fusion and decreases in fission. In combined fusion and fission, mitochondria size can increase and decrease. To qualify as a fusion, fission, or combined fusion and fission event, the level of size change must be significantly higher than the level of size fluctuation of individual separated mitochondria (Fig. 1B). On average, we observed 11 ± 4.2 (n = 3 experiments) fusion and fission events during the 40 minutes of imaging within the region of interest (ROI) (Fig. 1C). The two different modes of mitochondrial fusion identified in our study, namely fusion as well as combined fusion and fission, were consistent with findings of a previous study in cultured H9c2 cells^25^. Specifically, the fusion mode in our study was similar to the “complete fusion” mode in H9c2 cells^25^, and the combined fusion and fission mode in our study was similar to the “transient fusion” (“kiss-and-run”) mode^25^. However, significant mitochondrial size changes were detected in combined fusion and fission in our study, suggesting involvement of both fusion and fission machineries.

Out of the total of 35 fusion and fission events we observed, 27 were between stationary and moving mitochondria while the remaining 8 were between moving mitochondria. Normalized by the number of stationary mitochondria, this gave a total fusion and fission rate of 0.05 per mitochondrion per minute, similar to reported rates in the axon of primary mouse^26^ and rat^27^ neurons. Because the imaging region in our experiments was limited, we could only follow fast moving mitochondria for a short period of time. Therefore, our method likely underestimated the fusion and fission events between moving mitochondria.

### Validation of computational image analysis based detection of fusion and fission using photobleaching and fluorescence recovery

To independently validate our computational image analysis based detection of fusion and fission, we developed a photobleaching and fluorescence recovery assay (see Materials and Methods, supplementary Fig. S5). We first reduced intensities of mitochondria within a selected region by ~80% using photobleaching. We then monitored the intensity recovery of stationary mitochondria for fusion detection and analysis (Fig. 1D). We found that 66 ± 32% (n = 5 experiments) of stationary mitochondria exhibited intensity recovery by 39 ± 20% (n = 25 mitochondria) within 55 minutes post photobleaching. Recovery of mitochondrial intensity occurred abruptly and always coincided with passing of other moving mitochondria (Fig. 1D; supplementary Fig. S5). We therefore concluded that the recovery was due to mitochondrial fusion rather than gradual uptake of soluble mito-GFP from the axonal cytoplasm.

Out of the 23 observed recovery events, we identified 6 from fusion and 17 from combined fusion and fission (supplementary Fig. S6; supplementary Movies S6-7). However, our assay could only detect fusion as well as combined fusion and fission between stationary and moving mitochondria, but not between moving mitochondria. The numbers of fusion as well as combined fusion and fission detected were consistent with the numbers of such events between stationary and moving mitochondria detected by our computational image analysis (Fig. 1E). In addition, the size changes of mitochondria identified were consistent with the size change criterion used in our computational image analysis (Fig. 1F). Overall, the photobleaching and fluorescence recovery analysis validated the computational image analysis of fusion and fission based on mitochondrial size changes.

### Knockdown of dOpa1 caused significant reduction and imbalance of mitochondrial fusion and fission within the axon

To investigate the relations between mitochondrial fusion/fission and spatial distribution, we knocked down dOpa1, a mitochondrial inner membrane fusion protein in Drosophila^22^, using an RNA-interference strain dOpa1RNAi. We validated dOpa1 knockdown efficiency in constitutive elav-Gal4 promotor driven pan-neuronal expression of dOpa1RNAi in larval brains using western blots (supplementary Fig. S7). Using our computational image analysis method for fusion and fission detection, we characterized the frequency of fusion and fission events after dOpa1 knockdown (SG26 > dOpa1RNAi). We detected no fusion or combined fusion and fission events (Fig. 1C). The frequency of mitochondrial fission was also reduced significantly (Fig. 1C). Overall, downregulation of dOpa1 resulted in significant reduction and imbalance of mitochondrial fusion and fission within the axon.

### Knockdown of dOpa1 impaired mitochondrial transport

**Figure 2:**
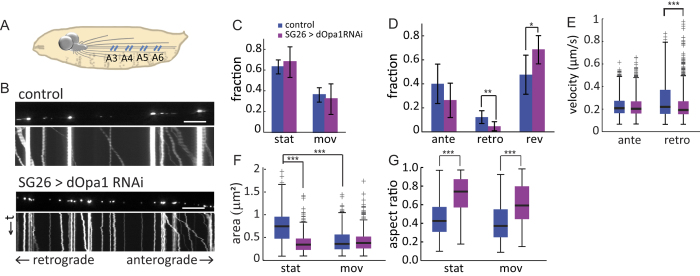
Effects of dOpa1 knockdown on motility and morphology of axonal mitochondria. (**A**) Time-lapse movies of mitochondria were collected in four axon regions (blue lines), corresponding to and named after abdominal segment A3, A4, A5 and A6, respectively. (**B**) Representative first frames and kymographs of movies from control and dOpa1 knockdown larvae. Scale bars: 10 μm. (**C**) Comparison of fractions of stationary and moving mitochondria in control versus dOpa1 knockdown axons in p-values: stationary, p = 0.42; moving: p = 0.52. Sample size: control, 1326 mitochondria from n = 8 axons; SG26 > dOpa1RNAi, 1694 mitochondria from n = 7 axons. (**D**) Comparison of fractions of different types of moving mitochondria in control versus dOpa1 knockdown axons in p-values: anterograde, p = 0.11; retrograde, p = 0.0078; reversing: p = 0.015. Error bars in (**C**,**D**) indicate SD. (**E**) Boxplot of mitochondrial velocities. dOpa1 knockdown significantly reduced retrograde segmental velocity (control, n = 947; SG26 > dOpa1RNAi, n = 1651; permutation t-test, p = 4.1 × 10^−20^), but did not affect anterograde segmental velocity (control, n = 2513; SG26 > dOpa1RNAi, n = 3138; permutation t-test, p = 0.47). (**F**) Boxplot of mitochondrial sizes in control versus dOpa1 knockdown axons: Stationary: control, 0.73 ± 0.33 μm^2^, n = 685; SG26 > dOpa1RNAi, 0.37 ± 0.17 μm^2^, n = 855; p = 1.4 × 10^−130^; Moving: control, 0.42 ± 0.24 μm^2^, n = 685; SG26 > dOpa1RNAi, 0.42 ± 0.23 μm^2^, n = 542; p = 0.48. In control, stationary mitochondria were significantly larger than moving mitochondria (p = 3.6 × 10^−54^). Under dOpa1 knockdown, stationary mitochondria became smaller than moving mitochondria (p = 5.1 × 10^−6^). (**G**) Boxplot of mitochondrial aspect ratios in control versus dOpa1 knockdown axons: Stationary: control, 0.46 ± 0.20; SG26 > dOpa1RNAi, 0.71 ± 0.19; p = 7.4 × 10^−112^; Moving: control, 0.42 ± 0.20; SG26 > dOpa1RNAi, 0.61 ± 0.21, p = 8.8 × 10^−43^. In both control and dOpa1 knockdown, stationary mitochondria were slightly but significantly more circular than moving mitochondria (control: p = 0.0002; SG26 > dOpa1RNAi, p = 1.2 × 10^−18^).

To test whether dOpa1 downregulation also affected mitochondrial transport in the axon, we collected time-lapse movies at 5 frames per second for 1 minute each in regions A3-A6 (Fig. 2A) and compared mitochondrial movement in control and dOpa1 knockdown (SG26 > dOpa1RNAi) larvae (see Materials and Methods; Fig. 2B). Movement and morphological changes of individual mitochondria were tracked as previously described^28^ (see Materials and Methods). We found that in control larvae, ~64% of the population to be stationary and ~36% to be moving (Fig. 2C,D). The average anterograde segmental velocity (see Materials and Methods) was 0.23 ± 0.08 μm/sec, and the average retrograde segmental velocity was 0.28 ± 0.16 μm/sec, consistent with previous reports^29^,^30^,^31^(Fig. 2E).

Knockdown of dOpa1 did not alter the overall fraction of stationary and moving mitochondria (Fig. 2C). However, within the moving population, the fraction of retrograde mitochondria was significantly reduced and the fraction of reversing mitochondria was mildly increased (Fig. 2D). Knockdown of dOpa1 had no significant effect on the average anterograde segmental velocity compared to control, but significantly reduced the average retrograde segmental velocity (Fig. 2E).

### Knockdown of dOpa1 changed morphology of stationary and moving mitochondria differentially in the axon

Because dOpa1 knockdown dramatically altered the balance between fusion and fission within the axon, we examined the morphology of axonal mitochondria. Within the axon of control larvae, the sizes of stationary and moving mitochondria differed significantly: the average size of stationary mitochondria was ~77% larger than that of moving ones (Fig. 2F). We found that overall, dOpa1 downregulation in Drosophila led to fragmentation of mitochondria within the axon, consistent with previous findings in cultured primary cortical neurons^18^. However, only stationary mitochondria were affected, as indicated by the reduction in their average area, not moving mitochondria (Fig. 2F). Consequently, stationary mitochondria became ~20% smaller than moving ones under dOpa1 knockdown (Fig. 2F). Both stationary mitochondria and moving mitochondria became significantly more circular under dOpa1 knockdown, as indicated by their higher average aspect ratios (Fig. 2G), consistent with previous findings in primary cortical neurons^18^.

Because we characterized sizes and aspect ratios of axonal mitochondria in their 2D wide-field microscopy images while actual mitochondria have 3D geometry, we checked and confirmed that their 2D images properly represented their actual 3D geometry (supplementary Fig. S8). Collectively, our data showed that stationary and moving axonal mitochondria differed significantly in size in control larvae and that their morphology, characterized in size and aspect-ratio, were changed differentially by dOpa1 knockdown. Stationary mitochondria became more severely fragmented under dOpa1 knockdown than moving mitochondria.

### Knockdown of dOpa1 changed spatial distributions of axonal mitochondria

**Figure 3:**
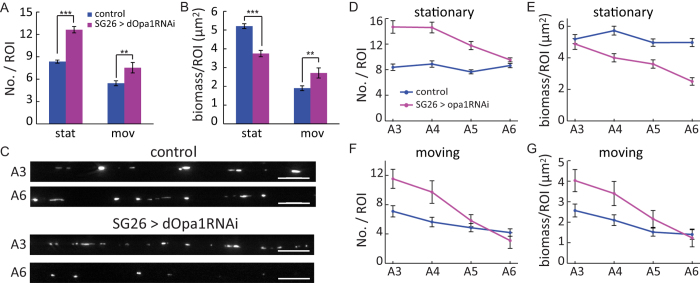
Downregulation of dOpa1 changed spatial distribution of axonal mitochondria. (**A**) Average number of mitochondria per ROI in control versus dOpa1 knockdown axons (control, n = 96 larvae; SG26 > dOpa1RNAi, n = 84 larvae). p-values: stationary, p = 7.4 × 10^−17^; moving, p = 0.0053; (**B**) Average biomass of mitochondria per ROI in control versus dOpa1 knockdown axons. p-values: stationary, p = 7.4 × 10^−11^; moving, p = 0.0064; Error bars in (**A**,**B**) indicate Standard Error of the Mean (SEM). (**C**) Representative images of mitochondria in A3 and A6 in wild-type and dOpa1 knockdown axons. Scale bars: 10 μm. Sample sizes in (**D**–**G**), control, n = 24; dOpaRNAi, n = 21. (**D**) Average number of stationary mitochondria in A3-A6. The regional difference was not significant (ANOVA, p = 0.18) in control, but was significant (p = 3.0 × 10^−6^) under dOpa1 knockdown. p-values for pairwise region comparison: A3, p = 3.9 × 10^−7^; A4, p = 3.6 × 10^−7^; A5, p = 2.1 × 10^−7^; A6, p = 0.06. (**E**) Average biomass of stationary mitochondria in A3-A6. Regional difference (ANOVA): control, p = 0.15; SG26 > dOpa1RNAi, p = 8 × 10^−7^. p-values for pairwise region comparison: A3, p = 0.49; A4, p = 5.9 × 10^−5^; A5, p = 3.6 × 10^−4^; A6, p = 1.5 × 10^−8^. (**F**) Average number of moving mitochondria in A3-A6 (regional difference: control, p = 0.012; SG26 > dOpa1RNAi, p = 0.0001). p-values for pairwise region comparison: A3, p = 0.004; A4, p = 0.0058; A5, p = 0.27; A6, p = 0.80). (**G**) Average biomass of moving mitochondria in A3-A6 (regional difference: control, p = 0.0055; SG26 > dOpa1RNAi, p = 0.0007). p-values for pairwise region comparison: A3, p = 0.02; A4, p = 0.021; A5, p = 0.12; A6, p = 0.78). Error bars in (D-G) indicate SEM.

We have thus far analyzed the role of dOpa1 in mediating mitochondrial fusion and fission as well as transport and morphology through knockdown. To investigate the relations between mitochondrial fusion/fission and spatial distribution, we first examined mitochondrial number and biomass (total sum of area) at the ensemble level by pooling data from all ROIs. Because stationary and moving mitochondria differed significantly in their morphology (Fig. 2F,G), we analyzed them separately. Overall, dOpa1 knockdown significantly increased the number of stationary mitochondria and significantly decreased their biomass (Fig. 3A,B). It also mildly but significantly increased the number and biomass of moving mitochondria (Fig. 3A,B).

We then examined the number and biomass of mitochondria in each of the four selected regions A3-A6, which spanned a distance of ~1600 μm (Figs 2A and 3C). In wild-type larvae, the number and biomass of stationary mitochondria were uniformly distributed (Fig. 3D,E). Under dOpa1 knockdown, the number and biomass of stationary mitochondria exhibited a distinct spatial pattern of progressive decrease towards the distal regions (Fig. 3D,E). Specifically, knockdown of dOpa1 significantly increased the number of stationary mitochondria in the proximal region A3 as well as middle regions A4-A5, but not in the distal region A6 (Fig. 3D). In contrast, the biomass of stationary mitochondria remained unchanged in the proximal region A3 but was significantly reduced in all other regions (Fig. 3E). Overall, the reduction of mitochondrial biomass in the middle and distal regions indicated that dOpa1 downregulation caused progressive loss of stationary mitochondria towards the distal regions.

For moving mitochondria, dOpa1 downregulation did not change the overall trend of decrease in number and biomass from region A3 to region A6 but the gradient became substantially steeper (Fig. 3F,G). The number of moving mitochondria decreased progressively from region A3 to region A6 in both control and dOpa1 knockdown larvae. However, the number of moving mitochondria was increased by ~50% under dOpa1 knockdown in the proximal region A3, but was largely unchanged in the distal region A6 (Fig. 3F). Overall, the percentage of decrease in the number of moving mitochondria from region A3 to region A6 was 27.5% in control, compared to 77.6% under dOpa1 knockdown. Unlike stationary mitochondria, the biomass of moving mitochondria also increased in regions A3-A4 under dOpa1 knockdown (Fig. 3G), but not in regions A5-A6. The percentage of decrease in the biomass of moving mitochondria from region A3 to region A6 was 36.1% in control, compared to 65.2% under dOpa1 knockdown. Taken together, our data revealed that in addition to disrupting inner membrane fusion, knockdown of dOpa1 changed spatial distribution patterns of stationary and moving mitochondria differentially, resulting in distinct spatial gradients of progressive decrease towards the distal regions.

### Milton knockdown caused similar transport velocity impairment as dOpa1 knockdown but different changes to mitochondrial spatial distribution

**Figure 4:**
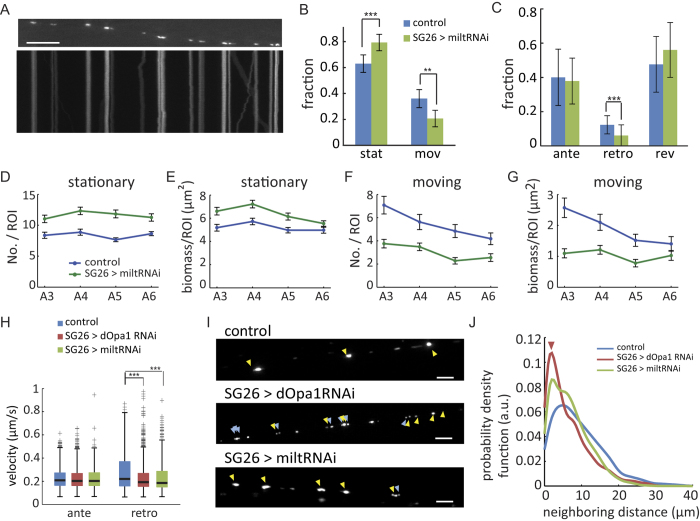
Axonal mitochondrial motility and spatial distributions under Milton knockdown. (**A**) First frame (upper panel) and kymograph (lower panel) of a time-lapse movie of axonal mitochondria under Milton knockdown. Scale bar: 10 μm. (**B**–**H**) Same control data as in Figs 1 and 2. (**B**) Comparison of fraction of stationary and moving mitochondria in control versus Milton knockdown in p-values. Stationary: control, n = 8 larvae; SG26 > miltRNAi, n = 15 larvae. p = 1.1 × 10^−5^. Moving: p = 2.8 × 10^−5^. (**C**) Comparison of fractions of different types of moving mitochondria in p-values: anterograde, p = 0.70; retrograde: p = 0.003, reversing: p = 0.25. Error bars in (**B**–**C**) indicate SD. Sample sizes in (**D**–**G**), control, n = 24; miltRNAi, n = 30. (**D**,**E**) No regional difference in the number of stationary mitochondria and decrease in the biomass under Milton knockdown (ANOVA, No./ROI, p = 0.43; biomass/ROI, p = 0.0023) (**F**,**G**) No regional difference in the number and biomass moving mitochondria under Milton knockdown (ANOVA, No./ROI, p = 0.11; biomass/ROI, p = 0.69). Error bars in (**D**–**G**) indicate SEM. (**H**) Neither dOpa1 knockdown nor Milton knockdown affected anterograde velocity (SG26 > miltRNAi (n = 1857), 0.22 ± 0.08 μm/s, permutation t-test, p = 0.057). Milton knockdown significantly reduced retrograde velocity (SG26 > miltRNAi (n = 478), 0.23 ± 0.14 μm/s, permutation t-test p = 1.1 × 10^−6^). (**I**) Representative frames showing distribution of stationary mitochondria. Yellow arrows: mitochondria remaining stationary during the entire movie. Blue arrows: mitochondria remaining stationary for > 5 minutes. Scale bars: 5 μm. (**J**) Comparison of distance between neighboring stationary mitochondria. The probability density function was calculated using kernel density estimation (see Materials and Methods).

Mitochondrial transport plays an important role in mediating spatial distribution of axonal mitochondria^24^,^32^,^33^,^34^. Since dOpa1 knockdown impaired mitochondrial transport (Fig. 2E), this raised the question whether changes to spatial distribution of axonal mitochondria under dOpa1 knockdown were merely a secondary effect of mitochondrial motility impairment. To test this hypothesis, we knocked down Milton, a mitochondrial adaptor for kinesin-1^23^,^24^, using RNA-interference (SG26 > miltRNAi) and analyzed transport, morphology and spatial distribution of axonal mitochondria (Fig. 4A). Milton is required for axonal transport of mitochondria in Drosophila^23^,^24^,^31^. Its downregulation significantly increased the fraction of stationary mitochondria, and significantly decreased the fraction of moving mitochondria (Fig. 4A,B). Within the moving population, the fraction of retrograde mitochondria was reduced under Milton knockdown (Fig. 4C). Similar as dOpa1 knockdown, Milton knockdown did not change the anterograde velocities of axonal mitochondrial transport (Fig. 4H; supplementary Fig. S11A). Also, similar as dOpa1 knockdown, Milton knockdown significantly reduced retrograde velocities of axonal mitochondrial transport (Fig. 4H; supplementary Fig. S11B). However, sizes of either stationary or moving mitochondria remained unchanged (stationary: 0.76 ± 0.36 μm^2^, n = 1011, p = 0.081; moving, 0.39 ± 0.22 μm^2^, n = 314, p = 0.072).

Both dOpa1 knockdown and Milton knockdown increased mitochondrial density in axons to a similar level, as characterized by the average number of mitochondria (NO./ROI, SG26 > dOpa1RNAi, 12.6 ± 1.8, n = 7; SG26 > miltRNAi, 11.6 ± 2.4, n = 15; p = 0.32). But they affected the spatial distribution of axonal mitochondria differentially (compare Fig. 3D–G vs Fig. 4D–G). Milton downregulation increased the number and the biomass of stationary mitochondria in regions A3-A6 (Fig. 4D–E) but decreased the number and the biomass of moving mitochondria in these regions (Fig. 4F–G). The number and the biomass of stationary mitochondria remained uniformly distributed under Milton knockdown (Fig. 4D). However, the gentle decreases in the number and biomass of moving mitochondria in control were eliminated in Milton knockdown (Fig. 4F–G).

To further investigate the spatial distribution of axonal mitochondria, we calculated the distances between neighboring stationary mitochondria. We found that they formed aggregates under both dOpa1 knockdown and Milton knockdown (Fig. 4I–J), as revealed by their reduced distances, but spacing between stationary mitochondria was further reduced under Milton knockdown compared to dOpa1 knockdown (Fig. 4J). The aggregation of stationary mitochondria observed under dOpa1 knockdown was consistent with reported aggregation of mitochondria in retinal ganglion cells with OPA1 deficiency^35^. Taken together, our data showed that Milton knockdown reduced retrograde velocities of axonal mitochondrial transport similarly as dOpa1 knockdown but changed the spatial distributions of axonal mitochondria differently. Therefore, the changed spatial distributions of axonal mitochondria under dOpa1 knockdown were not merely a secondary effect of mitochondrial motility impairment. Instead, they resulted primarily from the disruption of inner membrane fusion. Differential changes to mitochondrial spatial distributions under Milton knockdown also indicated that mitochondrial motility impairment under dOpa1 knockdown played a secondary role in causing the spatial distribution changes.

### Knockdown of dOp1a caused fragmentation of mitochondria before disorganization of cristae

**Figure 5:**
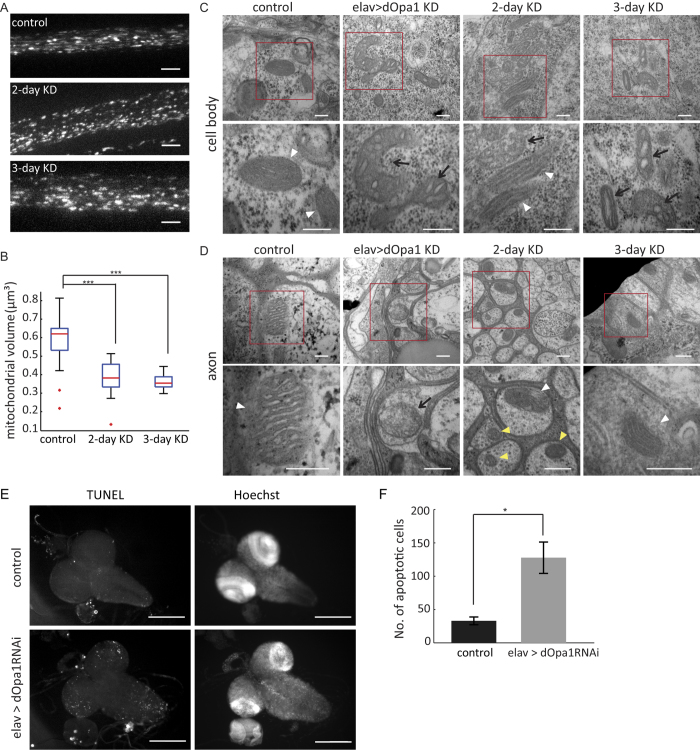
Knockdown of dOpa1 resulted in fragmentation of axonal mitochondria before substantial disorganization of cristae. (**A**) maximum projections of confocal z-stacks of mito-GFP under different conditions (control, induced knockdown for 2 days (2-day KD) and induced knockdown for 3 days (3-day KD)). Scale bars: 5 μm. (**B**) Boxplot of measured 3D volumes of mitochondria. The 3D volumes of mitochondria were significantly reduced in 2/3-day KD segmental nerve axons compared to control, but the volume changes were not significant between 2-day to 3-day knockdown. (control, 0.60 ± 0.1 μm^3^, n = 50; 2-day KD, 0.38 ± 0.09 μm^3^, n = 19; 3-day KD, 0.36 ± 0.04 μm^3^, n = 24; p_2-day KD_ = 7 × 10^−11^; p_3-day KD_ = 3.8 × 10^−16^). (**C**,**D**) Representative TEM images of mitochondria in ventral ganglia cell bodies (**C**) and cross sections of segmental nerves (**D**), respectively. Lower panels show enlarged views of the red box areas of corresponding upper panels. White arrowheads: mitochondria with normal cristae. Yellow arrowheads: smaller mitochondria. Black arrows: mitochondria with wider and vesicular cristae. Scale bars: 200nm. (**E**) Representative TUNEL staining (left) and Hoechst staining (right) images of larval brain. Scale bars: 200 μm. (**F**) L3 larvae with pan-neuronal dOpa1 knockdown exhibited increased but sporadic cell death in CNS and ventral ganglia (control, n = 3; elav > dopa1RNAi, n = 6; p = 0.02). Error bars indicate SEM.

Previous studies showed that loss of OPA1 resulted in disorganization of mitochondrial cristae, which released cytochrome c and induced cell apoptosis^36^,^37^. To determine whether changes to mitochondrial morphology observed under dOpa1 knockdown were a secondary effect of mitochondrial cristae disorganization and cell apoptosis, we knocked down dOpa1 at different developmental stages using a RU486 inducible elav-Gal4 driver (switch-elav > dOpa1RNAi)^38^,^39^ and characterized mitochondrial morphology and ultrastructure in segmental nerves of third instar larvae using confocal fluorescence microscopy and transmission electron microscopy (TEM) (Supplementary Information). We induced dOpa1 knockdown at early 2^nd^ or early 3^rd^ larval stage for 3 or 2 days, respectively. We found that loss of dOpa1 caused significant fragmentation of axonal mitochondria within two days of knockdown (Fig. 5A,B). We also observed a mixture of mitochondria with normal cristae and widened cristae in neuronal cell bodies after 2 days of induced knockdown (Fig. 5C). But mitochondrial cristae were largely intact in axons under induced knockdown of dOpa1 (Fig. 5D). Larvae with induced dOpa1 knockdown at early 2^nd^ or early 3^rd^ survived until late pupation or adult day 1.

Mitochondrial cristae became vesicular in both the cell body and the axon of larval neurons under constitutive pan-neuronal knockdown (elav > dOpa1 RNAi) (Fig. 5C,D). Because pan-neuronal dOpa1 knockdown led to mitochondrial cristae disorganization and animal death at L3 (3^rd^ larval) stage, we examined neuronal cell death in CNS and ventral ganglia L3 larvae using TUNEL staining. L3 larvae with pan-neuronal dOpa1 knockdown exhibited increased sporadic cell death in CNS and ventral ganglia (Fig. 5E,F), but no bulk neuronal cell death in their brains (Fig. 5E). These results indicated that dOpa1 has conserved functions in maintaining cristae organization and morphology as its mammalian ortholog^40^. Taken together, our data indicated that the observed axonal mitochondrial fragmentation under dOpa1 knockdown was unlikely a secondary effect of cristae disorganization and cell apoptosis. Intact cristae structure is important to mitochondrial health and function. Our data did not exclude the possibility that disruption of cristae organization under dOpa1 knockdown may also contribute to the changed spatial distributions of axonal mitochondria.

### Disruption of inner membrane fusion reduced axon length of cultured primary larval neurons without changing mitochondrial membrane potential

**Figure 6:**
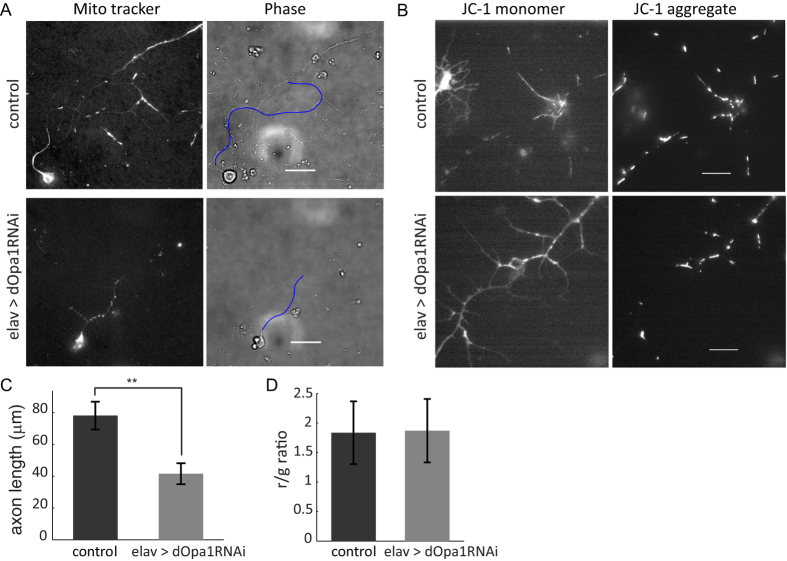
Knockdown of dOpa1 impaired axon growth without changing mitochondrial membrane potential. (**A**) Representative images of cultured primary neurons on DIV3. Left panels: fluorescence images of mitochondria labeled by Mitotracker Red; Right panels: phase contrast images of the same neurons. Blue lines indicate traced axons. Scale bars: 20 μm. (**B**) Representative JC1 staining images of cultured primary larval neurons. Left panels: JC-1 monomer; Right panels: JC-1 aggregate. Scale bars: 10 μm. (**C**) Knockdown of dOpa1 impaired axon growth (control, n = 36; elav > opa1RNAi, n = 18; p = 0.008). Error bars indicate SEM. (**D**) Knockdown of dOpa1 did not affect mitochondrial membrane potential, measured by the red (JC-1 aggregate) to green (JC-1 monomer) ratio (control, n = 117; elav > dopa1RNAi, n = 125; p = 0.7). Error bars indicate SD.

We observed that disruption of mitochondrial inner membrane fusion resulted in changes to spatial distribution of axonal mitochondria. To check developmental outcomes that may be connected with such changes, we examined axon growth in cultured primary larval neurons. Neurons from dOpa1 knockdown larvae grew significantly shorter axons after 3-day in culture (Fig. 6A,C). To check physiological outcomes that may be connected with such changes, we examined mitochondrial membrane potential in cultured primary larval neurons using JC-1 staining. We found that mitochondrial membrane potentials were similar in control and dOpa1 knockdown neurons (Fig. 6B,D). In summary, changes to the spatial distribution of axonal mitochondria due to disruption of inner membrane fusion led to impaired axon growth but had no significant effect on mitochondrial membrane potential.

## Discussion

Although individual mitochondria within the axon may appear as discrete compartments, they interconnect through fusion and fission as well as transport and anchoring to form a dynamic network. This network is distributed spatially to fulfill changing needs at different locations. Proper spatial distribution of axonal mitochondria has been shown, for example, to be essential for axon branching^34^,^41^, synaptic functions^13^,^42^,^43^ and a variety of other neuronal activities^10^,^44^. In this study, we have focused on addressing a basic question regarding the axonal mitochondrial network, namely whether, and if so how, local fusion and fission of individual mitochondria affect their global distribution.

We found that inner membrane fusion, which occurred locally between individual mitochondria, mediated the global distribution of mitochondria within the axon. In wild-type Drosophila larvae, spatial distribution of stationary and moving axonal mitochondria followed distinct patterns (Fig. 3D–G). Disruption of inner membrane fusion by dOpa1 knockdown not only caused dramatic imbalance between fusion and fission and fragmentation of individual mitochondria (Figs 1C and 2F) but also changed their spatial distribution patterns, resulting in progressive loss of both stationary and moving mitochondria along the axon towards distal axon regions (Fig. 3D–G). Consistent with this result, disruption of mitochondrial outer membrane fusion by knocking down Marf (Drosophila ortholog Mfn2) resulted in similar progressive loss of stationary and moving mitochondria along the axon (supplementary Fig. S9). On the other hand, knockdown of mitochondrial motor adaptor Milton impaired retrograde velocities of mitochondrial transport similarly as dOpa1 knockdown but changed the spatial distribution of axonal mitochondria differently (Fig. 4D–G). Together, these results showed that the changes to the spatial distributions of axonal mitochondria under dOpa1 knockdown were caused primarily by disruption of inner membrane fusion and that impairment of mitochondrial transport under dOpa1 knockdown played a secondary role in causing these changes. Therefore, local inner membrane fusion plays an important role in mediating the global spatial distribution of mitochondria.

Our study is in agreement with a growing number of studies suggesting that, in addition to mediating local mitochondrial content exchange or transport-docking, fusion and fission are involved in regulating the global organization of the mitochondrial network^7^,^14^,^16^, although direct analysis of the relations between local fusion/fission and the global organization of the mitochondrial network was lacking in previous studies. Our data reveals direct connections and quantitative relations between mitochondrial inner membrane fusion and spatial distribution. However, a limitation of our assay is that it is restricted to the specific group of neurons in which SG26 Gal4 is expressed. Further studies should examine relations between mitochondrial fusion/fission and spatial distribution in different groups of neurons.

A unique challenge facing neurons is to sustain functionally competent mitochondria over extended distances. Indeed, neurons are known to be particularly vulnerable to dysfunction of mitochondria and mutations of mitochondrial proteins^45^,^46^,^47^. Since the soma of neurons is the primary site for biogenesis and degradation of mitochondria^48^, a basic question regarding axonal mitochondria is how they stay functionally competent and renew themselves while being far away from the neuronal cell body. Our data supports the hypothesis that axonal mitochondria replenish themselves through fusion with moving ones passing by and through fission to discard their damaged portion^46^. Specifically, we found that within the axon of Drosophila larval neurons, stationary mitochondria underwent fusion and fission regularly with moving mitochondria (Fig. 1A,C). They were also much larger than moving ones but became more fragmented under dOpa1 knockdown (Fig. 2F). Together, our data suggests differential roles of stationary and moving mitochondria: while stationary mitochondria fulfill metabolic and functional needs of their local areas, moving mitochondria support stationary mitochondria by renewing their content through fusion/fission. Furthermore, moving mitochondria can move to areas where new needs arise and settle down as stationary mitochondria. Our data supports the quality control model of axonal mitochondria previously proposed^47^ but does not rule out the possibility that populations of stationary and moving mitochondria interchange through direct switching between their motion states by a transport-docking mechanism^5^,^13^,^49^.

**Figure 7:**
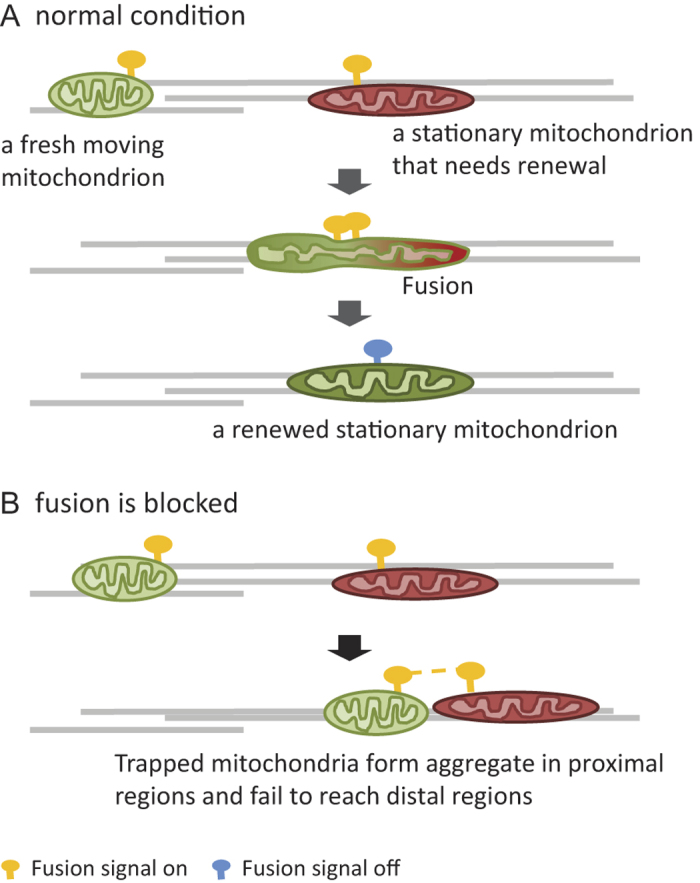
A model of how local inner membrane fusion mediates the global spatial distribution of axonal mitochondria. (**A**) A stationary mitochondrion undergoes fusion and fission with a moving mitochondrion for renewal. The fusion signal is inactivated after the renewal is successfully completed. (**B**) Disruption of inner membrane fusion prevents completion of renewal and therefore inactivation of the fusion signal. Some moving mitochondria are trapped by stationary mitochondria with activated fusion signal. They form aggregate in regions close to the soma and increasingly fail to reach distal axonal regions.

Based on our data, we propose a model of how the spatial distribution of axonal mitochondria is maintained in healthy neurons and how it is changed by disruption of inner membrane fusion (Fig. 7). We conjecture that anchored stationary mitochondria activate a fusion signal when renewal is needed (Fig. 7A). The fusion signal retains some of the passing mitochondria to engage in fusion and fission. Successful completion of fusion and fission inactivates the signal (Fig. 7A), allowing moving mitochondria to simply pass by. When inner membrane fusion is disrupted by OPA1 knockdown, the fusion signal of stationary mitochondria can no longer be inactivated because fusion cannot be completed. Cristae disorganization under dOpa1 knockdown may also interfere with the successful fusion of mitochondria. The stationary mitochondria close to the soma and with activated fusion signal will retain increasing numbers of moving mitochondria so that fewer moving mitochondria can reach more distal regions (Fig. 7B). This results in a gradual accumulation of stationary mitochondria close to the soma and progressive loss of mitochondria along the axon towards distal synaptic terminals. The loss of mitochondria in distal regions eventually leads to neurodegeneration.

## Materials and Methods

### Statistical analysis

Unless specified otherwise, mean values were shown in the form of mean ± Standard Deviation (SD). Statistical analysis was performed using MATLAB (MathWorks) and R. Distribution of data was first checked as previously described^28^ using model-based clustering R package *mclust*^50^. For data that largely followed a single normal distribution, their means and distributions were compared using two-sample, two-sided Student’s t-tests and Kolmogorov-Smirnov tests, respectively. For data that followed non-normal distributions, we first assessed difference between their means using non-parametric permutation t-tests^51^. We also assessed their differences by comparing their distributions in terms of their medians using Wilcoxon rank-sum tests. ANOVA test was used for comparing spatial distributions of mitochondria. The probability density function of spacing distances between neighboring stationary mitochondria was calculated by kernel density estimation using MATLAB built in function *ksdensity*.

### Genetics

The following stocks were purchased from Bloomington Drosophila Stock Center: *elav-Gal4, UAS-dOpa1RNAi* (TRiP.HMS00349), *UAS-miltRNAi* (TRiP.JF03022), UAS-marfRNAi (TRiP.HMC03883), *switch-elav Gal4* and *UAS-mito-GFP/CyO*. The single neuron Gal4 driver *pGAL4 SG26-1*^52^ was a gift from Dr. Lawrence Goldstein (University of California San Diego). Stocks were maintained at 25 °C, except that crosses of UAS transgene lines and *pGAL4 SG26-1* were set up at 29 °C for single neuron expression. To study mitochondrial dynamics in the axon of single neurons, we first crossed male *UAS-mito-GFP/CyO* with female *pGAL4 SG26-1*. *UAS-mito-GFP/*+*; pGAL4 SG26-1/*+ was used as the control. To study mitochondrial dynamics under dOpa1, Marf, or Milton knockdown, we further crossed the male *UAS-mito-GFP/*+*; pGAL4 SG26-1/*+ with virgin *UAS-dOpa1RNAi, UAS-marfRNAi*, or *UAS-miltRNAi*, respectively.

### Time-lapse imaging

Drosophila third instar larvae were dissected in calcium free HL3 buffer (in nM; 128 NaCl, 1 ethylene glycol tetraacetic acid (EGTA), 4 MgCl2, 2 KCl, 5 HEPES, and 36 sucrose) to expose the segmental nerves as previously described^28^. Segmental nerves immersed in HL3 buffer were inverted onto a #1.5 coverslip (Fisher Scientific) for imaging. Time-lapse movies were collected immediately after dissection at room temperature on a Nikon Eclipse Ti-E inverted microscope with a CoolSNAP HQ2 camera (Photometric) and a 100×/1.40 NA oil objective lens. The effective pixel size was 0.0645 μm. Mito-GFP was imaged using a FITC filter set. Movies were collected from four equally spaced regions, corresponding to and named after the stereotypical larval body segments A3~A6, along each larval axon. For motility and morphology analysis, each time-lapse movie was collected at 5 frames per second for 1 minute. For each region of each animal, at least 2~3 time-lapse movies were collected to reduce data variation. For fusion and fission analysis, each time-lapse movie was collected at 30 frames per minute for 40 minutes. No photo toxicity and little photobleaching were observed during imaging.

### Computational image analysis of mitochondrial transport and morphological changes

Movement of individual mitochondria in collected time-lapse movies was tracked largely as previously described for vesicles^28^. Specifically, image registration was first performed to correct for sample drift^53^. Then individual mitochondria were segmented^54^ and tracked using their centroids as their positions in a semi-automatic fashion^28^.

Mitochondrial movement was analyzed largely as previously described for vesicles^28^. Briefly, from trajectory of each mitochondrion, its frame to frame displacement d_i_ was calculated to determine its direction of instantaneous movement. The instantaneous velocity v_i_ and its deviation dev_i_ were calculated for each mitochondrion using a 7 frame sliding window, with their directions defined by the sign of 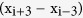, i.e.,


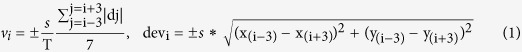


where s denotes the effective pixel size and T denotes the duration of each frame. If both instantaneous velocity and sliding window deviation of a mitochondrion were small 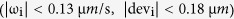 , we identified the mitochondrion as pausing. The threshold values were determined from manual identification of pause events followed by calculation of their instantaneous velocity and sliding window deviation. When a mitochondrion remained pausing for more than 6 seconds, we classified it as a pause event. Each mitochondrial trajectory was divided into segments separated by pause events. The direction of each segment was determined by the sign of its instantaneous velocity. If the sign of instantaneous velocities reversed and then remained for a continued period of time (>6 sec) within a segment, we identified the event as a reversal and divided the segment at the reversal^28^.

The segmental velocity of a mitochondrion is its mean velocity within a consistently unidirectional trajectory segment^28^. The average velocity of stationary mitochondria was previously reported to be less than 0.012 μm/s^49^. We set two criteria to detect a stationary mitochondrion. First, its maximum instantaneous velocity within a trajectory should be no greater than 0.2 μm/s. Second, its maximum deviation within a trajectory in the 1 min time-lapse movie should be less than 20 pixels (1.29 μm). Again, these threshold values were determined from manual identification of stationary mitochondria followed by calculation of their instantaneous velocity and maximum deviation. For morphology analysis, we used only trajectory segments in which there was no overlap between individual mitochondria to ensure that only well separated mitochondria were analyzed.

For each genetic background (e.g. control, dOpa1 knockdown, Milton knockdown), analyzed data from different experiments were pooled into a single dataset for analysis. In particular, control data was collected for each experiment. Analysis results of data from all control experiments were pooled into a single control dataset. Quality of the control velocity data was further checked using bootstrap tests (see Supplementary Information; supplementary Fig. S12).

### Identifying mitochondrial fusion and fission by computational image analysis

Time-lapse movies were collected in an axon region ~500 μm away from the ventral ganglia in both control (SG26 > mitoGFP) and dOpa1 knockdown (SG26 > dOpa1RNAi) larvae. The width of this region of interest (ROI) was about 80 μm. We identified mitochondria fusion and fission in the time-lapse movies by combining computational image analysis with visual verification. Specifically, after recovering mitochondrial trajectories by software tracking, fusion and fission events were identified based on three criteria. First, there must be overlap between trajectories of the mitochondria involved. Such overlap can be very brief when two mitochondria move past each other. Or it can be very long when two mitochondria stay together for a long time. Second, the size changes of mitochondria before and after fusion or fission must be greater than the detection threshold. Sizes of individual separated mitochondria within the axon remained generally stable over time, with an average standard deviation of ~0.034 μm^2^ (n = 1115; supplementary Fig. S10). We set the threshold of size change of individual mitochondria for detecting fusion and fission to be 0.1 μm^2^, three times the average standard deviation. Third, there must be clear visual cues to avoid ambiguity in interpretation (see discussion below). When all these criteria were met, we classified the event as a fusion or fission event.

We identified three types of fusion and fission events, which we refer to as fusion, fission, and combined fusion and fission, respectively. A *fusion* is an event in which two or more mother mitochondria come together and fuse into one daughter mitochondrion (supplementary Fig. S2; Movie S1, S2). In this case, the size difference between the largest mother mitochondrion and the daughter mitochondrion must be greater than the threshold. A *fission* is an event in which a mother mitochondrion splits into two or more smaller daughter mitochondria (supplementary Fig. S3; Movie S3, S4). Again, the size differences between the mother mitochondrion and the largest daughter mitochondrion must be greater than the threshold. A *combined fusion and fission* is an event in which two or more mother mitochondria come together and produce two or more daughter mitochondria (supplementary Fig. S4; Movie S5). For size analysis, mother mitochondria and daughter mitochondria are sorted base on their sizes. Then larger mother mitochondria are paired with larger daughter mitochondria, and smaller mother mitochondria are paired with smaller daughter mitochondrion. Size differences between at least one pair must be greater than the threshold.

Other than size changes, visual cues were used to differentiate fusion/fission from simple trajectory overlapping. In the case of fusion or fission between moving mitochondria, we usually observed mitochondria moving together after fusion or before fission (supplementary Fig. S2A, S3A; Movies S1, S3). In the case of fusion or fission between stationary and moving mitochondria, we usually observed dynamic reshaping of the stationary mitochondria (supplementary Fig. S2B, S3B; Movies S2, S4).

There are cases when a very small moving mitochondrion and a stationary mitochondrion overlapped in their trajectories for a long period of time. This occurred, for example, when a moving mitochondrion entered a stationary mitochondrion without an identifiable outgoing trajectory and without significant size changes. Because such events could not be differentiated from simple trajectory overlapping, we excluded them from our analysis. The occurrence rate of such events was low in our movies, generally no more than once over the 40 minutes of imaging in the region of interest.

### Identifying mitochondrial fusion by photobleaching and fluorescence recovery

Drosophila third instar larvae were dissected as described above. Experiments were conducted on control mito-GFP larvae (SG26 > mitoGFP) within a region ~500 μm away from the ventral ganglia. We first collected 5 minutes of time-lapse movies at 30 frames per minute using a 488 nm laser (Agilent) at 1% power for fluorescence excitation. We then photobleached the same region using the laser at full power (1.8 kW/cm^2^) for less than 1 minute. Mitochondria intensity was reduced by ~80% after photobleaching. Immediately after photobleaching, we collected time-lapse movies at 30 frames per minute for 55 minutes using the laser at 2% power for fluorescence excitation. Time-lapse movies were collected using a Nikon Eclipse Ti-E inverted microscope with a CoolSNAP HQ2 camera (Photometric) and a 100×/1.49 NA oil objective lens. In total, we imaged axonal mitochondria in the selected region for 60 minutes, 5 minutes before photobleaching and 55 minutes after photobleaching. Stationary mitochondria were visually identified after photobleaching by their residual intensity (supplementary Fig. S5). The distribution of full-intensity moving mitochondria recovered in 10–15 minutes in the bleached region. Thus the effective imaging duration for characterizing fusion events in this experiment was comparable with other experiments in this study.

We tracked and characterized intensity of each stationary mitochondrion within a 5 × 10 pixel (pixel size is 0.06 μm) box. We reported the following normalized intensity for individual mitochondria:


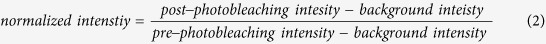


The pre-photobleaching intensity and post-photobleaching intensity were mean intensities within the boxed area. We used only intensity values when there was no transient intensity fluctuation due to crossing of moving mitochondria.

We did not observe gradual mitochondrial intensity recovery and thus ruled out the possibility of fluorescence recovery by uptake of soluble mito-GFP. Mitochondrial intensity increases abruptly following fusion or combined fusion and fission in post-photobleaching imaging (Fig. 1D). We identified fusion (supplementary Fig. S6B; Movie S7) or combined fusion and fission (supplementary Fig. S6A; Movie S6) based on intensity recovery of stationary mitochondria. We characterized morphological changes of mitochondria based on their segmentation (Fig. 1F).

## Additional Information

**How to cite this article**: Yu, Y. *et al.* Inner membrane fusion mediates spatial distribution of axonal mitochondria. *Sci. Rep.* **6**, 18981; doi: 10.1038/srep18981 (2016).

## Supplementary Material

Supplementary Information

Supplementary Video S1

Supplementary Video S2

Supplementary Video S3

Supplementary Video S4

Supplementary Video S5

Supplementary Video S6

Supplementary Video S7
