# Supplementary material for: Inner membrane fusion mediates spatial distribution of axonal mitochondria
Source: Sci Rep. 2016 Jan 8;6:18981. doi: 10.1038/srep18981 (PMC4705540; doi:10.1038/srep18981)
Supplement: Supplementary Information [file srep18981-s1.pdf]

Supplementary information

Inner membrane fusion mediates spatial distribution of axonal mitochondria

Yiyi Yu, Hao-Chih Lee, Kuan-Chieh Chen, Joseph Suhan, Minhua Qiu, Qinle Ba, Ge Yang

Supplementary material and methods

Inducible knockdown

We knocked down dOpa1 at different developmental stages of Drosophila using RU486 inducible elav-Gal4 driver (switch-elav > dOpa1RNAi)[1,2] and characterized mitochondrial morphology in segmental nerves of third instar larvae. Expression of UAS transgenes reached the highest level in ~21 hours after feeding[2]. For inducible gene expression, crosses were carried out on normal food, and 2nd or early 3rd instar larvae were collected and transferred for feeding of food containing 15ug/ml RU486. We fed 2nd or early 3rd instar larvae for 3 or 2 days, respectively, before wandering 3rd instar larvae were collected for TUNEL staining, in vivo imaging, or transmission electron microscopy (TEM). Flies under induced dOpa1 knockdown died at late pupa stage or on adult day 1 or day 2. For imaging mito-GFP under induced dOpa1 knockdown, female switch-elav Gal4 was crossed with male *UAS-mito-GFP/CyO* to generate a control. Male *UAS-mito-GFP/+; switch-elav Gal4/+* was further crossed with *UAS-dOpa1RNAi* female to generate *UAS-mito-GFP/UAS-dOpa1RNAi; switch-elav Gal4/+*. For TEM analysis, *elav-Gal4* or *switch-elav Gal4* female was crossed with *UAS-dOpa1RNAi* male. Age-matched *UAS-dOpa1RNAi* larvae were used as a control.

Transmission electron microscopy

Drosophila third instar larvae were fixed in 3% paraformaldehyde with 1% glutaraldehyde at 4°C for 24 hours. After washing in 3 rounds of PBS, the samples were placed in a 1% osmium tetroxide solution buffered with PBS for one hour, followed by 3 round of

washing with water. The brain and nerve fibers were dissected from the body. The samples were dehydrated in a series of ethanol solutions of increasing concentration (50%, 70%, 95%, and 100%). Propylene oxide was used as a transitional solvent, and the samples were placed in a 1:1 mixture of Spurr resin and propylene oxide, and stored overnight in a desiccator. The following day, the Spurr and propylene oxide mixtures were removed and replaced with 100% Spurr resin. The samples were infiltrated with the Spurr resin for an additional 8 hours, placed in flat embedding molds, and polymerized for 48 hours at 60ºC. The samples were re-embedded in Spurr resin in an orientation with the cerebral hemispheres at the base of the embedding mold. Thick sections of 2 microns were taken for approximately 50 microns. At that distance, thin (100nm) sections were cut using a DDK diamond knife on a Reichert-Jung Ultracut E ulatramicrotome. The sections were stained with lead citrate for 1 minute, and were observed for section and stain quality. If the quality was adequate, the samples were sectioned an additional 50 microns deeper into the specimen, and another set of thin sections were collected and stained. This procedure was continued until the nerve fibers had been sectioned at a distance of several hundred microns from the brain. The grids were viewed on a Hitachi H-7100 transmission electron microscope (Hitachi High Technologies) operating at 75 keV. Digital images were collected using an AMT Advantage 10 CCD Camera System (Advanced Microscopy Techniques) and inspected using NIH ImageJ software.

3D confocal microscopy

To characterize mitochondrial morphology in the thick bundle of segmental nerves, we used 3D confocal microscopy. Three dimensional z-stacks of mito-GFP within the segmental nerve bundle of control and inducible dOpa1 knockdown larvae were collected on a spinning

disk confocal microscope with an EMCCD camera (Andor Technology). The effective pixel size was 0.105 µm in the x and y dimension, and 0.2 µm in the z dimension. Mitochondria in each image were segmented by custom software in 2D using the difference of Gaussian filter followed by adaptive thresholding, as previously described[3]. The 3D volume of each mitochondrion was determined by stacking their 2D segmentations.

TUNEL staining

Brains of third instar larvae containing intact CNS and ventral ganglia were dissected in standard HL3 media. Positive control was included in each round of assay. For each round and each genotype, 3~4 brains were collected in 500 µl tubes. Brains were fixed in 4% PFA in PBS overnight in 4 ºC and were washed in 0.3% Triton-X100, 0.1% sodium citrate (pH 6) for 1 hour. Then they were rinsed in PBS with 0.1% Triton-X100 (PBST) 3 times. Positive controls were treated with DNaseI (Sigma) for 10 mins. Brains were blocked with 5% BSA diluted in PBST for one hour at room temperature. TUNEL staining was carried out following manufacturer's protocol (Roche). Brains were incubated with TUNEL staining reagents for 2 hours at room temperature in darkness. After washing with PBST, brains were stained with 10ug/ml Hoechst 33342 (Invitrogen) for 1 hour. TUNEL signals were imaged using a Nikon Eclipse Ti-E inverted microscope with a CoolSNAP HQ2 camera (Photometric) and a 20× objective lens. We took 1.8 µm thick stacks through the whole sample. TUNEL positive signals were quantified from the maximal projection of collected z-stacks. Positive signals were detected by the Otsu segmentation function in ImageJ.

Primary larval neuron culture and mitochondrial staining

Third instar larval brains (including hemisphere and ventral ganglia) were dissected in Rinaldini's saline (NaCl, 137 mM; KCl, 2.68 mM; NaH2PO4, 0.36 mM; NaHCO3, 11.9 mM; glucose, 5.55 mM). Brains were treated with 0.2 mg/ml Collagenase I (Sigma) for 30 mins under room temperature and then mechanically dissociated using fire polished glass pipettes. Primary neurons were maintained in Schneider's Insect Medium (Invitrogen) supplemented with 10% fetal bovine serum (GIBCO) and 50 μg/ml insulin (Sigma).

To measure axon growth, primary neurons were treated with 500 nM mitoTracker Red for 5 mins on DIV3. Images were taken on a Nikon Eclipse TE2000-U inverted microscope with an Andor EMCCD camera and a 60×/1.40 NA oil objective lens. Axon arbor was imaged under DIC and mitochondria were imaged using a Cy3 filter set. Axons were traced manually. Axon traces were smoothed using polynomial fitting in MATLAB.

To measure mitochondrial membrane potential, primary larval neurons were treated with JC1 dye at 10ug/ml for 20 mins on DIV 3. JC1 dye was diluted in full culture media and centrifuged to remove pellet. Images were taken by a CoolSNAP HQ2 camera. JC-1 monomer (green channel) was imaged using a FITC filter set; while JC-1 aggregate (red channel) was imaged using a TRITC filter set. The intensity of the red emission is proportional to mitochondrial membrane potential. To measure the ratio of red/green intensity, we first used image segmentation after Gaussian filtering to identify individual mitochondria. We then calculated the ratio between red and green channel intensities for each identified mitochondrion.

Western blot analysis

Twenty brains of Drosophila third instar larvae were collected in ice cold PBS and homogenized in RIPA (Life Technologies) supplemented with PMSF (Sigma) and protease inhibitor cocktails (Sigma). Proteins were separated on 10% polyacrylamide gel by electrophoresis in Tris-Glycine buffer, and transferred to nitrocellulose membrane. Membranes were blocked in 5% non-fat milk for 1 hour at room temperature. Blocked membranes were blotted with mouse anti-GAPDH (Santa Cruz) or mouse anti-OPA1 (Abnova) overnight at 4 ºC. On the following day, after washing with 0.1% Tween TBS, membranes were blotted with HRP conjugated secondary antibody (Thermo). SuperSignal West Pico Substrate (Thermo) was used for detection. Membranes were imaged using a LAS-3000 imager (GE Healthcare).

Bootstrap testing of control velocity data

Control data were collected for each experiment. Their analysis results were pooled into a single dataset to ensure that the sample size was sufficiently large for reliable determination of statistical distribution of mitochondrial properties such as transport velocity, size, and aspect ratio. Specifically, a total of 8 control experiments were conducted, with an average of 12 time-lapse movies collected in each experiment. In total, 96 time-lapse movies were analyzed, and the analysis results were pooled into a single control dataset. To check whether the analysis of control data provided reliable and consistent results, bootstrap tests[4] were conducted on mitochondrial transport velocities. First, out of the 8 control experiments, a group of 7 experiments were randomly selected, and the transport velocities were pooled. Then, the velocities pooled from the 7 experiments were compared against the velocities pooled from all 8

experiments using permutation t-tests[4]. This bootstrap testing was repeated for all the possible combinations (i.e. C_8^7). It was found that in 100% (out of C_8^7) of the trials, the two dataset showed no significant difference (all p value > 0.05). This procedure was then repeated by randomly selecting and pooling data from 6, then 5, then 4 experiments. It was found that in 96% (out of C_8^6), 88% (out of C_8^5) and 80% (out of C_8^4), respectively, of the trials, there was no significant difference compared to results pooled from all 8 experiments. The complete sets of bootstrap testing results were summarized in Figure S12. Several observations can be drawn from the results. First, the velocity data pooled from all 8 experiments did not provide statistically different information compared to velocity data pooled from any 7 experiments. Thus the data pooled from the 8 experiments should be sufficient in determining the statistical distributions of mitochondrial velocities. It is unlikely that adding more experiments will provide statistically different information. Second, the velocity data from any combination of 7 experiments provided statistically the same information. It is unlikely that repeating the control experiments will provide statistically different information. Third, if the number of control experiments is fewer than 7, even pooling the data together cannot guarantee (i.e. with 100% probability) that it will accurately determine the statistical distribution of the data. For example, in the case of pooling data from 4 randomly selected control experiments, there is a ~20% probability that the pooled data will show a statistically different velocity distribution compared to the data pooled from all 8 experiments.

Supplementary figures

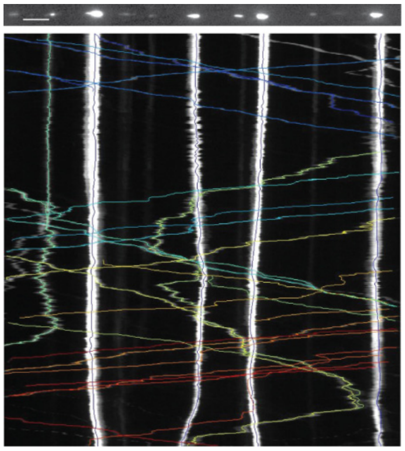

Fig. S1. Computer tracking of individual axonal mitochondria. First frame (upper panel) and kymograph (lower panel) of a representative 40 minutes movie of mito-GFP (SG26 > UAS mitoGFP) collected in a region ~500 µm away from the ventral ganglia of a wild-type larva. The frame rate was 30 frames per minute. Individual mitochondria were tracked as previously described[5] using custom software with some modifications. Recovered trajectories were randomly colored and overlaid onto the kymograph for inspection. Scale bar: 10 µm.

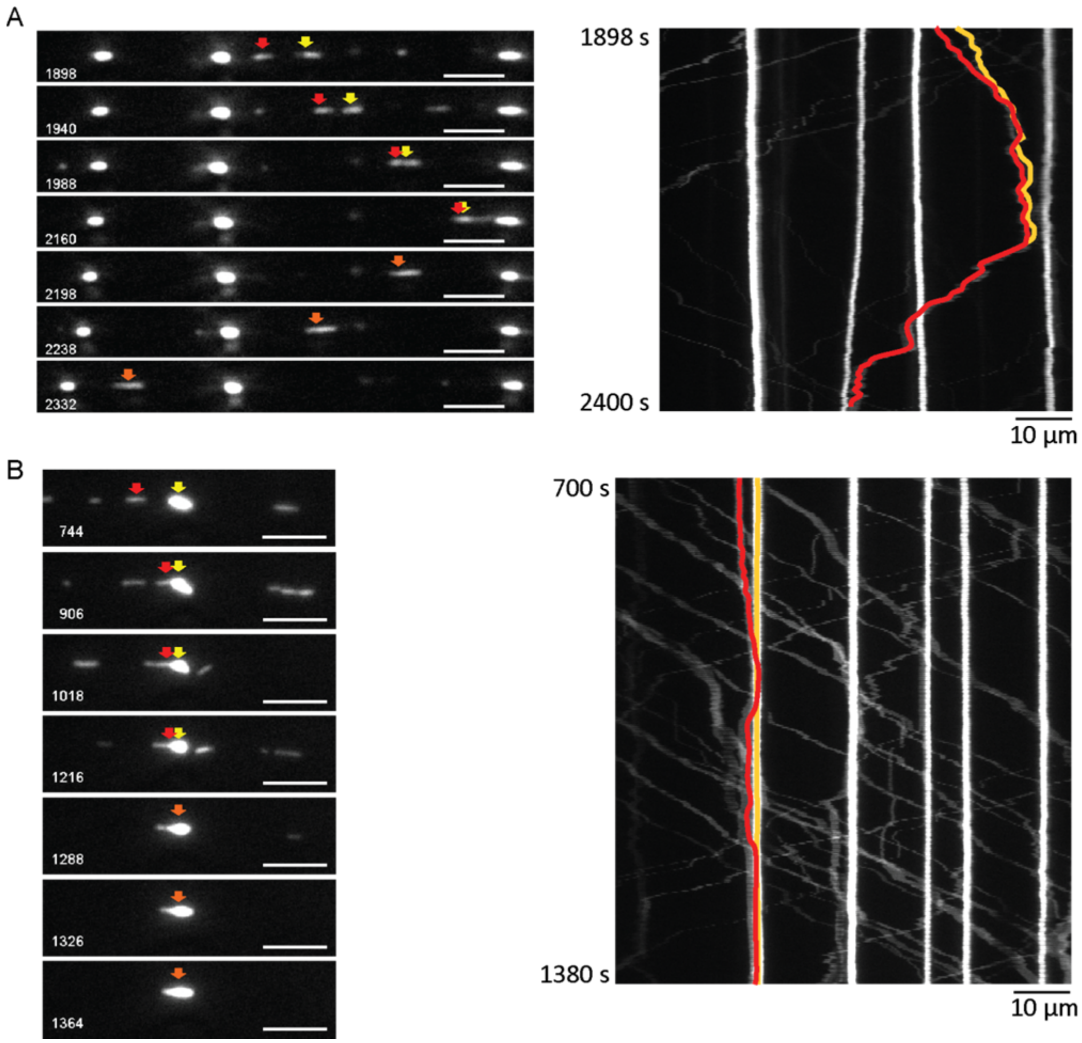

Fig. S2. Representative examples of mitochondrial fusion within the larval axon. (A) Fusion of two moving mitochondria. Left panel: selected frames from the time-lapse movie of the fusion event. The number in the lower left corner indicates time in seconds. Red and yellow arrows point to the two mother mitochondria, and orange arrows point to the daughter mitochondrion. Scale bars: 5 µm. Right panel: corresponding kymograph of trajectories of the two fusing mitochondria. For each mitochondrion, its arrow color in the left panel and trajectory color in the right panels were matched. The size of the larger moving mitochondria was increased by 0.51 μm^2 after fusion. The two initially separated mitochondria moved together after the fusion. This was identified as a visual cue in verification. (B) Fusion of a moving mitochondrion with a

stationary mitochondrion. Same panel layout and color scheme as in (A). Scale bars in the left panel: 5 μm. The size of the stationary mitochondrion was increased by 0.12 μm^2 after fusion. The stationary mitochondrion changed its shape dynamically in the fusion. This was identified as a visual cue in verification. Movies S1 and S2 are the corresponding time-lapse movies for (A) and (B), respectively.

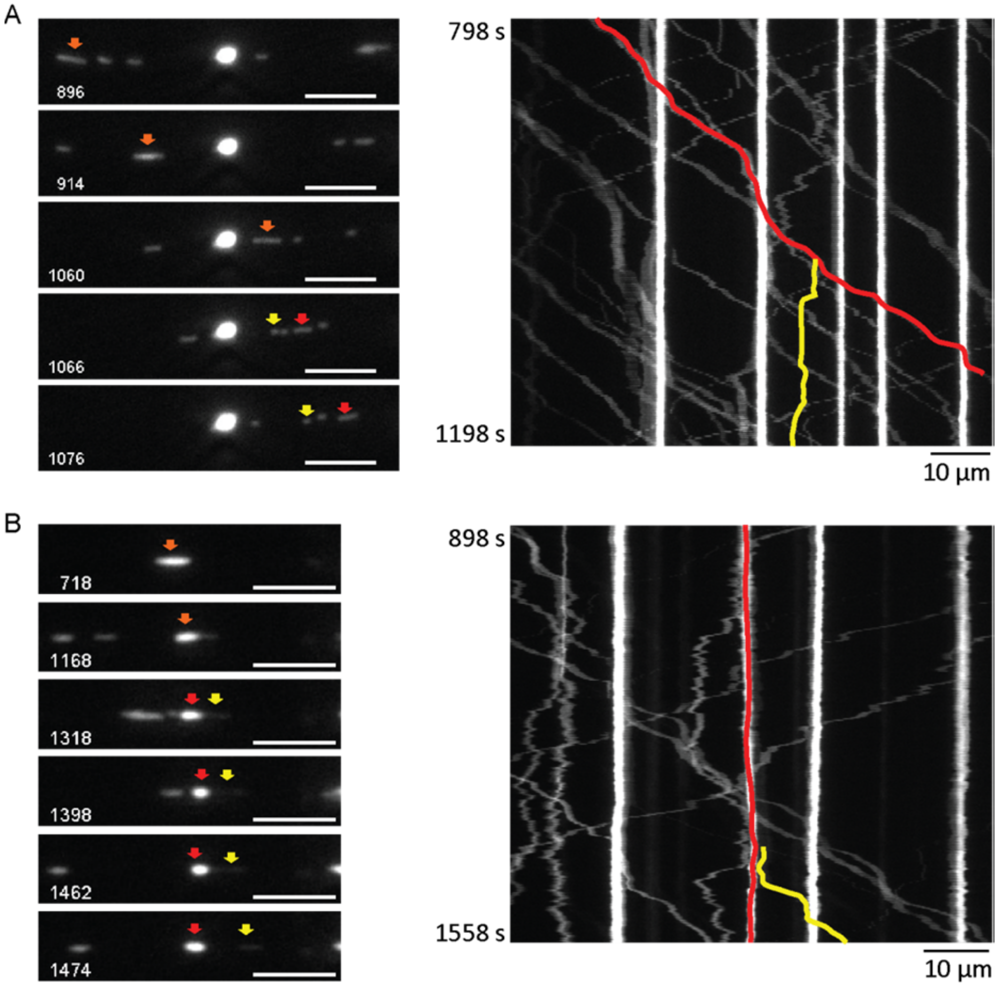

Fig. S3. Representative examples of mitochondrial fission within the larval axon. (A) Fission of a moving mitochondrion. Left panel: selected frames from the time-lapse movie of the fission event. The number in the lower left corner indicates time in seconds. Orange arrows point to the mother mitochondrion, and red and yellow arrows point to the two daughter mitochondria. The size decrease from the mother mitochondrion to the larger daughter mitochondrion was 0.17 μm^2. Scale bars: 5 µm. Right panel: corresponding kymograph of trajectories of the fission event. For each mitochondrion, its arrow color in the left panel and trajectory color in the right panels are matched. The trajectory of the mother mitochondrion split into two in the kymograph. This was identified as a visual cue in verification. (B) Fission of a stationary mitochondrion. Same

panel layout and color scheme as in (A). Scale bars in the left panel: 5 μm. The size of the stationary mitochondrion was decreased by 0.3 μm^2 after fission. The stationary mitochondrion exhibited substantial shape changes during fission. This was identified a visual cue in verification. Movies S3 and S4 are the corresponding time-lapse movies for (A) and (B), respectively.

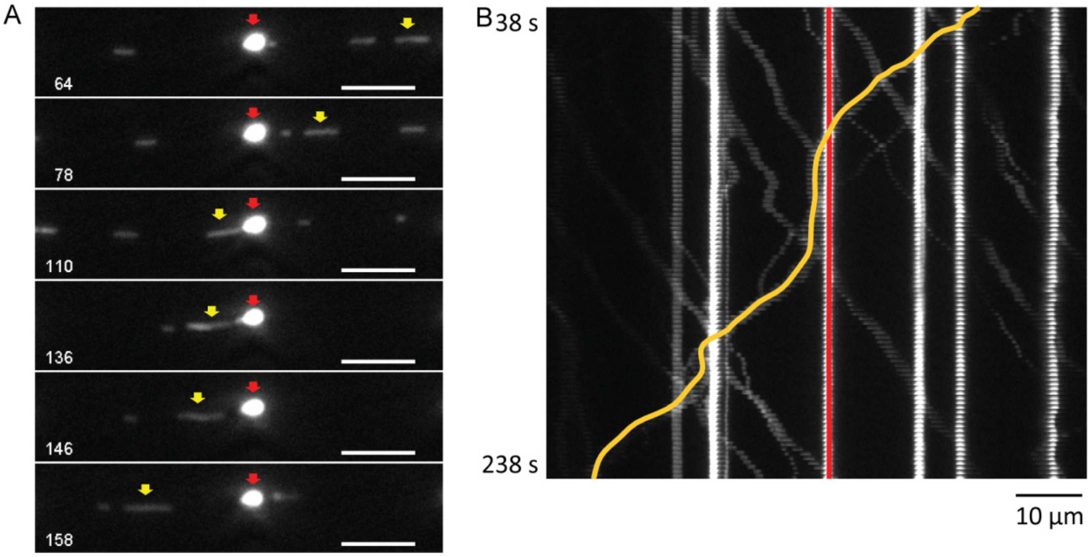

Fig. S4. A representative example of combined mitochondrial fusion and fission within the larval axon. (A) Selected frames from the time-lapse movie of a combined fusion and fission event between a stationary mitochondrion and a moving mitochondrion. The number in the lower left corner indicates time in seconds. Yellow arrows point to the moving mitochondrion, and red arrows point to the stationary mitochondrion. Scale bars: 5 µm. The size of the moving mitochondrion was decreased by 0.25 μm^2 by the combined fusion and fission. (B) Corresponding kymograph of trajectories of the combined fusion and fission event. For each mitochondrion, its arrow color in (A) and trajectory color in (B) were matched. Movie S5 is the corresponding time-lapse movie for (A).

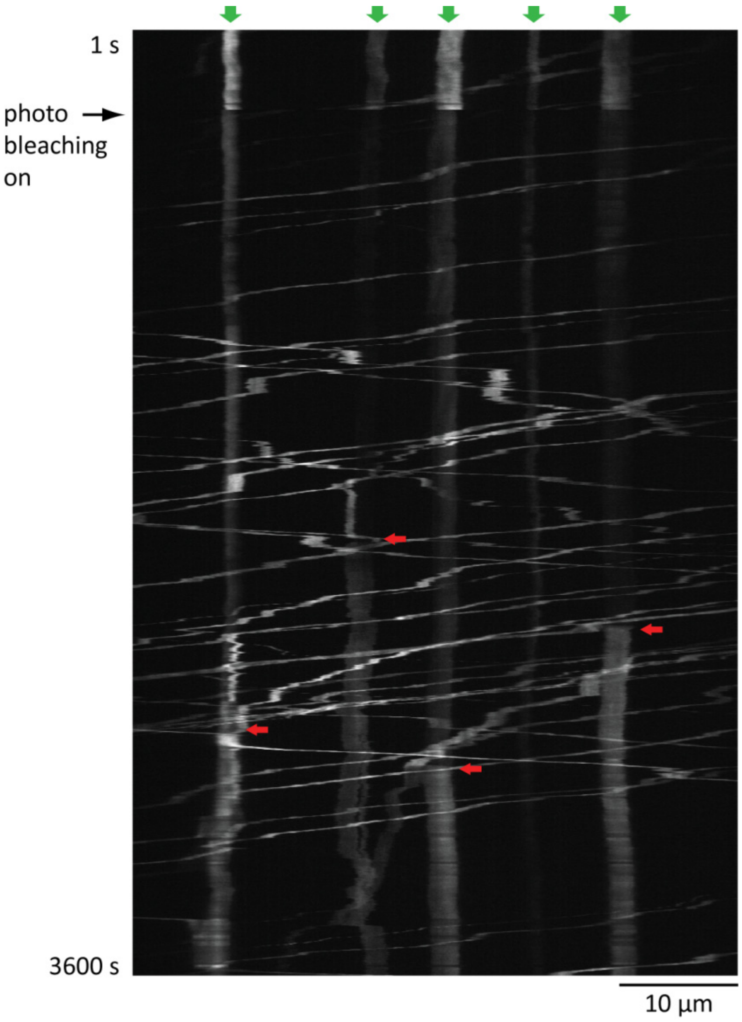

Fig. S5. Fluorescence recovery of stationary mitochondria after photobleaching. Kymograph of a representative time-lapse movie from a wild-type larva expressing mito-GFP (sg26 > UAS-mito-GFP). Five stationary mitochondria were marked by green arrows at the top. The intensity change of each mitochondrion was revealed by its corresponding trace in the kymograph. Four out of the five mitochondria showed intensity recovery, and the starting time points of their abrupt intensity increase were marked by red arrows. The average intensity recovery of the four mitochondria was 54 ± 23% (mean±SD, n = 4). No gradual intensity recovery indicative of soluble mito-GFP uptake was observed.

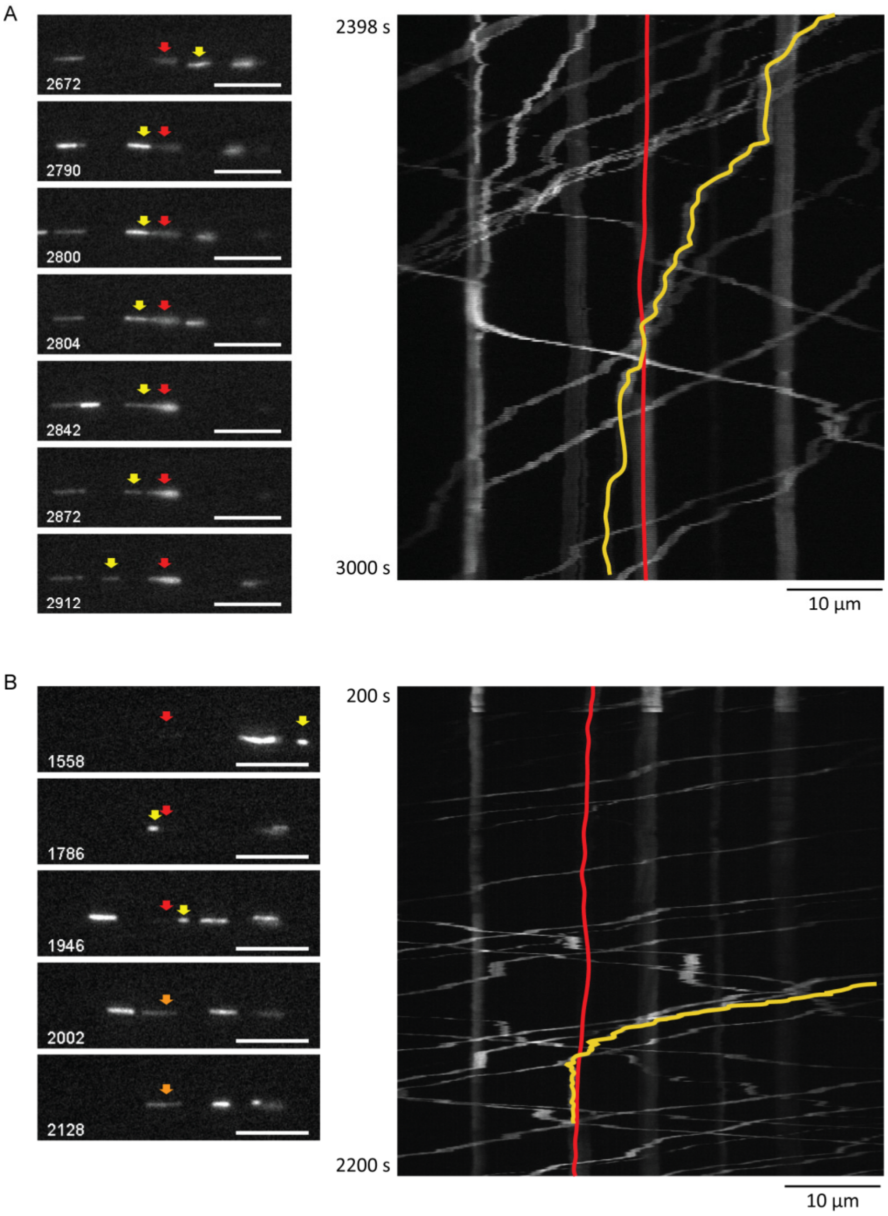

Fig. S6. Examples of mitochondrial fusion identified by photobleaching and fluorescence recovery. (A) Combined fusion and fission of a stationary mitochondrion (red arrow) with a moving mitochondrion (yellow arrow). Left panel: selected frames from the corresponding time-lapse movie of the event. The number in the lower left corner indicates time in seconds. The intensity of the stationary mitochondrion was recovered by ~34% after combined fusion and fission with the moving mitochondrion. The size of the moving mitochondrion was decreased by

0.37 μm^2 after the combined fusion and fission. Scale bars: 5 μm. Right panel: corresponding kymographs of trajectories of the combined fusion and fission event. For each mitochondrion, its arrow color in the left panel and trajectory color in the right panels were matched. (B) Fusion of a stationary mother mitochondrion (red arrows) with a moving mother mitochondrion (yellow arrow) into a daughter mitochondrion (orange arrows). Same panel layout as in (A). The intensity of the stationary mitochondrion was recovered by 70% after fusion with the moving mitochondrion. Movies S6 and S7 are the corresponding time-lapse movies for (A) and (B), respectively.

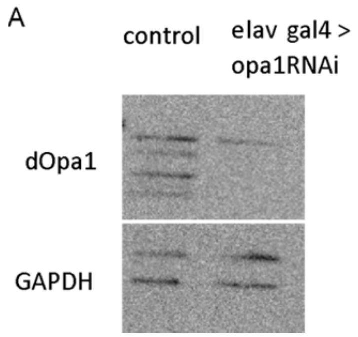

Fig. S7. Western blot analysis of dOpa1 protein levels in wild-type and pan-neuronal dOpa1 knockdown larvae. GAPDH was used as a loading control.

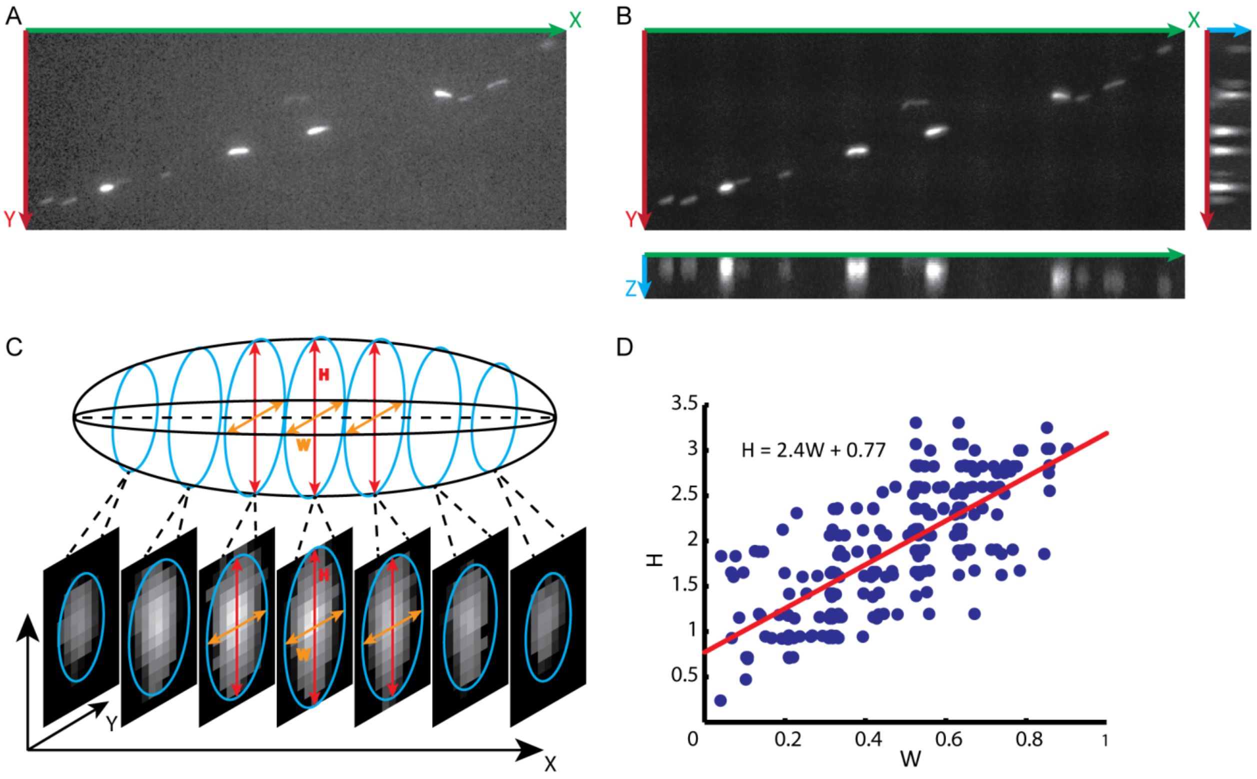

Fig. S8. 2D images of axonal mitochondria properly represent their 3D geometry. Actual axonal mitochondria are three-dimensional. This raised the question of whether the collected 2D wide-field images of individual mitochondria would properly represented their 3D geometry. To address this question, we collected 2D wide-field and 3D confocal microscopy images of the same group of mitochondria. We found that their 3D aspect ratio remained largely uniform along each mitochondrion within the axon. This indicated that the 2D area of each mitochondrion could be used to properly represent its size in 3D. (A) A 2D wide-field image of axonal mitochondria along an axon. (B) Maximum projection of a 3D confocal microscopy image stack of the same group of mitochondria shown in (A). Top panel: XY-plane projection. Left panel: XZ-plane projection. Bottom panel: YZ-plane project. (C) Upper panel: a cartoon illustrating the typical 3D geometry of an axonal mitochondrion. Lower panel: a series of YZ cross-section images of a mitochondrion. Effective pixel size along the Y and Z directions were 105 and 230 nm,

respectively. (D) A scatter plot of all pairs of height (H) and width (W) of sampled cross-sections of mitochondria. A total of 440 YZ-cross sections were uniformly sampled from 22 mitochondria. The scatter plot showed that their hight (H) and weight (W) were significantly correlated (correlation coefficient = 0.71), indicating a generally uniform aspect ratio along each mitochondrion. The red regression line shows the aspect ratio between H and W is ~2.4.

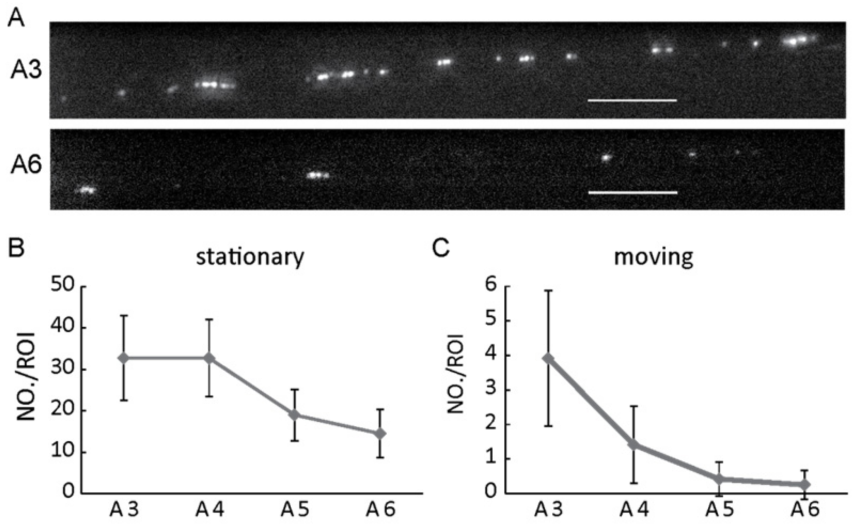

Fig. S9. Spatial distribution of axonal mitochondria under Marf knockdown. (A) Representative images from time-lapse movies of mitochondria in proximal region A3 and distal region A6 under Marf knockdown (sg26 > marfRNAi). Scale bars: 10 µm. (B, C) Marf knockdown led to gradual loss of stationary (p = 0.001; ANOVA, n = 6) and moving (p = 0.0008) mitochondria along the axon towards synaptic terminals. Error bars indicate SEM.

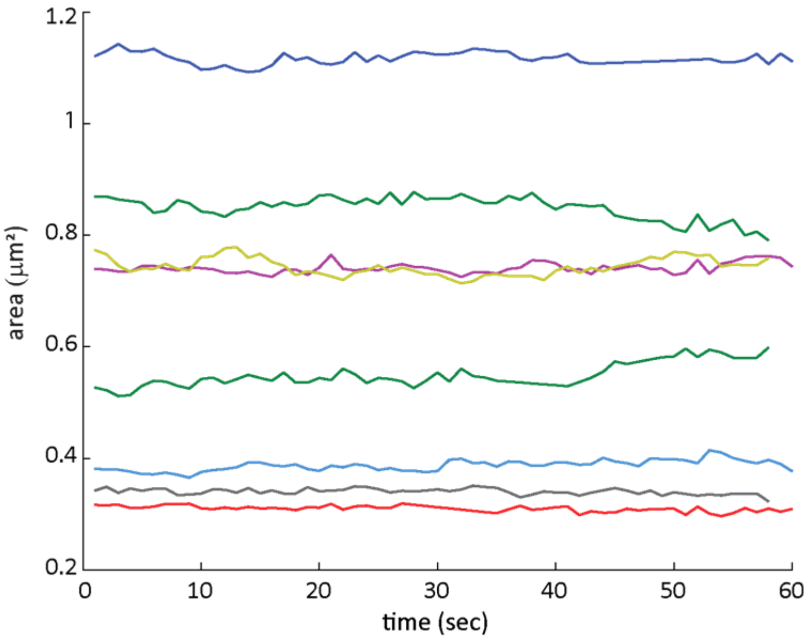

Fig. S10. Representative time series of size fluctuations of individual separated mitochondria within the larval axon. Sizes of individual separated mitochondria remained largely stable over time, with an average standard deviation of 0.034 μm^2 (n = 1115). Colors were randomly assigned to differentiate between different mitochondria.

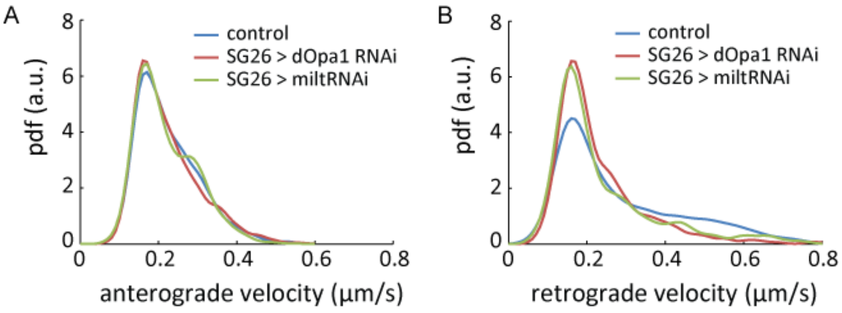

Fig. S11. Statistical distributions of mitochondria transport velocities under different genetic backgrounds. (A) The probability density functions (pdf's) of anterograde velocities under control, dOpa1 knockdown, and Milton knockdown were not significantly different (Wilcoxon rank-sum tests: control vs. dOpa1 knockdown, $p = 0.096$; control vs. Milton knockdown, $p = 0.082$; dOpa1 knockdown vs Milton knockdown, $p = 0.76$). (B) The pdf's of retrograde velocities under dOpa1 knockdown and Milton knockdown were significantly different from that of control (Wilcoxon rank-sum tests: control vs. dOpa1 knockdown, $p = 6.43\times10^{-11}$; control vs. Milton knockdown, $p = 1.12\times10^{-6}$; dOpa1 knockdown vs Milton knockdown, $p = 0.53$).

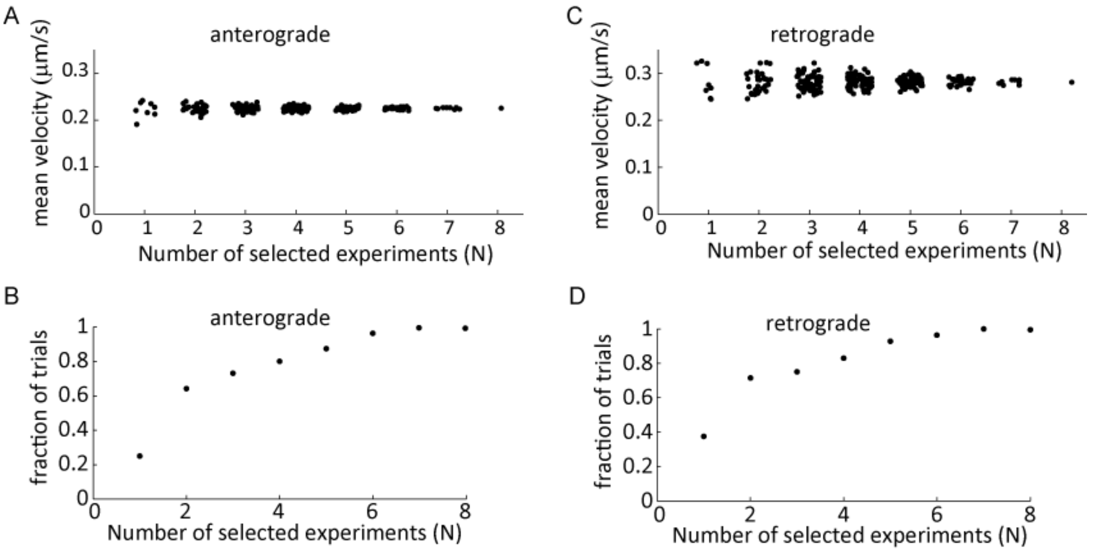

Fig. S12. Bootstrap testing of control velocity data. A total of 8 control experiments were conducted. Velocity data from N = 1 ~ 7 randomly selected control experiments were pooled into a single dataset and then compared against the velocity data pooled from all 8 experiments using permutation t-tests[4]. For each N, all combinations (i.e. C_8^N) were tested. (A, C) Mean anterograde velocities (A) and retrograde velocities (C) at different numbers of randomly selected experiments. Each dot represents the mean velocity of a randomly selected combination of experiments. (B, D) Fractions of trials (out of C_8^N) in which the pooled anterograde velocities (B) and retrograde velocities (D) showed no significant difference (permutation t-test p-value >=0.05) compared to the velocities pooled from all 8 experiments.

Supplementary videos

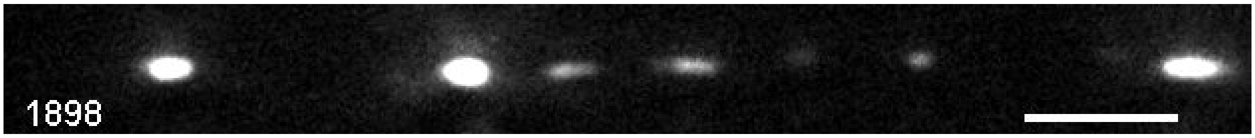

Movie S1. An example of fusion between two moving mitochondria, with selected frames shown in Fig. S2A. Scale bar: 5 µm

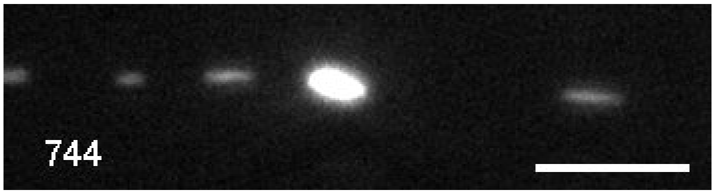

Movie S2. An example of fusion between a stationary mitochondrion and a moving mitochondrion, with selected frames shown in Fig. S2B. Scale bar: 5 µm

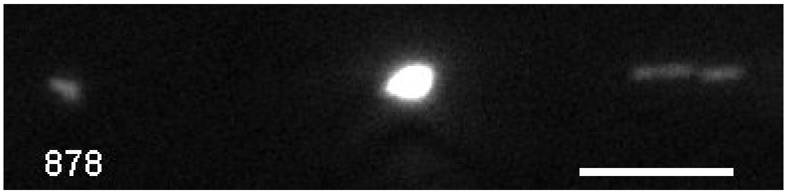

Movie S3. An example of fission of a moving mitochondrion, with selected frames shown in Fig. S3A. Scale bar: 5 µm

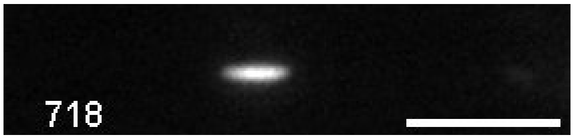

Movie S4. An example of fission of a stationary mitochondrion, with selected frames shown in Fig. S3B. Scale bar: 5 µm

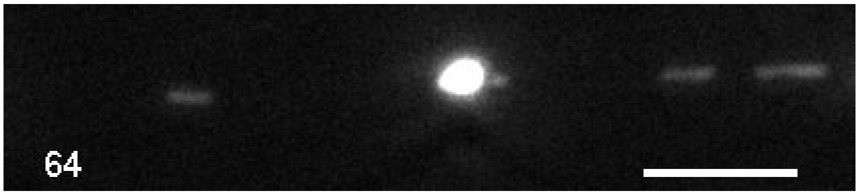

Movie S5. An example of a combined fusion and fission, with selected frames shown in Fig. S4. Scale bar: 5 µm

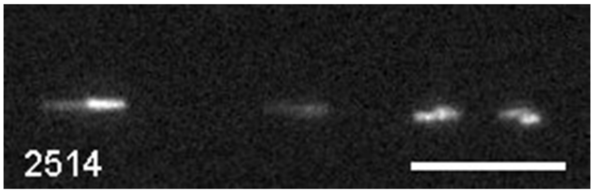

Movie S6. An example of fluorescence intensity recovery of a stationary mitochondrion after combined fusion and fission with a moving mitochondrion, with selected frames shown in Fig. S6A. Scale bar: 5 µm

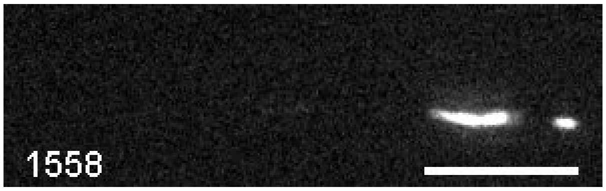

Movie S7. An example of fluorescence intensity recovery of a stationary mitochondrion after fusion with a moving mitochondrion, with selected frames shown in Fig. S6B. Scale bar: 5 µm

References

1. Nicholson, L. *et al.* Spatial and temporal control of gene expression in Drosophila using the inducible GeneSwitch GAL4 system. I. Screen for larval nervous system drivers. *Genetics* **178,** 215–234 (2008).

2. Osterwalder, T., Yoon, K. S., White, B. H. & Keshishian, H. A conditional tissue-specific transgene expression system using inducible GAL4. *Proc. Natl. Acad. Sci. U. S. A.* **98,** 12596–12601 (2001).

3. Chen, K.-C., Yu, Y., Li, R. & Lee, H.-C. Adaptive active-mask image segmentation for quantitative characterization of mitochondrial morphology. *IEEE Int. Conf. Image Process.* 2033–2036 (2012).

4. Moore, D. S., McCabe, G. P. & Craig, B. A. *Introduction to the Practice of Statistics*. (W. H. Freeman, 2009).

5. Reis, G. F. *et al.* Molecular motor function in axonal transport in vivo probed by genetic and computational analysis in Drosophila. *Mol. Biol. Cell* **23,** 1700–1714 (2012).
